# Grazers and Phytoplankton Growth in the Oceans: an Experimental and Evolutionary Perspective

**DOI:** 10.1371/journal.pone.0077349

**Published:** 2013-10-24

**Authors:** Simona Ratti, Andrew H. Knoll, Mario Giordano

**Affiliations:** 1 Dipartimento di Scienze della Vita e dell’Ambiente, Università Politecnica delle Marche, Ancona, Italy; 2 Department of Organismic and Evolutionary Biology, Harvard University, Cambridge, Massachusetts, United States of America; University of Melbourne, Australia

## Abstract

The taxonomic composition of phytoplankton responsible for primary production on continental shelves has changed episodically through Earth history. Geological correlations suggest that major changes in phytoplankton composition correspond in time to changes in grazing and seawater chemistry. Testing hypotheses that arise from these correlations requires experimentation, and so we carried out a series of experiments in which selected phytoplankton species were grown in treatments that differed with respect to the presence or absence of grazers as well as seawater chemistry. Both protistan (*Euplotes* sp.) and microarthropod (*Acartia tonsa*) grazers changed the growth dynamics and biochemical composition of the green alga *Tetraselmis suecica*, the diatom *Thalassiosira weissflogii*, and the cyanobacterium *Synechococcus* sp., increasing the specific growth rate and palatability of the eukaryotic algae, while decreasing or leaving unchanged both parameters in the cyanobacteria. *Synechococcus* (especially) and *Thalassiosira* produced toxins effective against the copepod, but ciliate growth was unaffected. *Acartia* induced a 4-6 fold increase of Si cell quota in the diatom, but *Euplotes* had no similar effect*.* The differential growth responses of the eukaryotic algae and cyanobacteria to ciliate grazing may help to explain the apparently coeval radiation of eukaryophagic protists and rise of eukaryotes to ecological prominence as primary producers in Neoproterozoic oceans. The experimental results suggest that phytoplankton responses to the later radiation of microarthropod grazers were clade-specific, and included changes in growth dynamics, toxin synthesis, encystment, and (in diatoms) enhanced Si uptake.

## Introduction

Microfossils, molecular biomarkers, and molecular clocks all indicate that the taxonomic composition of phytoplankton in continental shelf waters has changed episodically through Earth history [1]. In part, this reflects the timing of evolutionary innovation: cyanobacteria are older than the algae that incorporated them as primary endosymbionts, and red and green algae predate the haptophytes, alveolates and stramenopiles that gained photosynthesis via secondary endosymbiosis. But there must be more to the story because green algae did not rival photosynthetic bacteria as primary producers until hundreds of millions of years after chlorophytes first evolved [2]. And the evolutionary introduction of chlorophyll a+c algae into Mesozoic oceans did not by itself insure ecological dominance – some Chl a+c clades remain minor participants in the marine carbon cycle, and despite radiations in several algal clades, cyanobacteria persist as principal primary producers in many open ocean environments.

Today, the spatial distribution of phytoplankton mirrors the environmental heterogeneity of surface oceans [3], suggesting the possibility that observed long term trends in phytoplankton composition might find at least partial explanation in the changing nature of marine environments through time (e.g., [4]). In a previous paper [5], we reported an initial set of physiological experiments asking whether seawater chemistry might have favored different photosynthetic clades at different times. Seawater solutions were prepared with [SO_4_^2-^] that varied from 1 to 30 mM; sulphate was targeted because (1) the limited stoichiometric data available for phytoplankton suggest that modern shelf dominants – diatoms, coccolithophorids, and dinoflagellates – have higher S:C than green algae or cyanobacteria [6] – and (2) geochemical data suggest that seawater [SO_4_^2-^] has increased through time. Growth rates for the cyanobacterial and green algal strains used in this experiment were insensitive to [SO_4_^2-^], but the algae that have dominated shelf production over the past 100 million years, especially dinoflagellates and coccolithophorids, exhibited higher growth rates with increasing [SO_4_^2-^], at least up to levels inferred for the late Paleozoic to early Mesozoic oceans in which these groups first evolved. In direct competition experiments, using seawater designed to approximate the chemistry of Proterozoic, Paleozoic and modern oceans, diatoms outcompeted other algae in the modern seawater solution, but, consistent with paleontological data, green algae were superior competitors in the “Paleozoic” medium.

Such experiments, of course, leave residual uncertainty, as they are necessarily limited to a small number of taxa and are vulnerable to the charge that the biology of living algae owes more to recent physiological adaptation than it does to evolutionary constraint. Further testing is needed to establish differences among taxa with statistical rigor. Yet, the experimental results do fit predictions of the motivating hypothesis and so show that physiological experiments can, in principle, supplement ecological and evolutionary perspectives based on observation and modelling.

Here we revisit our simple experimental scheme to explore another potentially important aspect of phytoplankton evolution: response to grazing. Grazing plays a major role in structuring pelagic ecosystems [7]. Like the physical environment, grazing pressure varies spatially within modern oceans and has changed through time with innovations of phytoplankton grazing, first by protists and then by small metazoans. Here we explore the motivating hypothesis that phylogenetically distinct phytoplankton respond differentially to grazing in ways that might inform the geologically observed evolutionary history of marine phytoplankton. Once again, we have kept our experimental apparatus simple, growing a small number of phylogenetically distinct phytoplankton strains in the presence or absence of protistan and invertebrate grazers. The experiments were conducted under a range of sulphate abundances to ask whether there might be an interactive grazing x environmental chemistry effect. Our results lend preliminary experimental support to the hypothesis that grazers, as well as seawater chemistry, helped to shape the observed evolutionary history of shelf phytoplankton.

## Materials and Methods

### Cultures

Semi-continuous cultures of the cyanobacterium *Synechococcus* sp. (UTEX LB 2380), the green alga *Tetraselmis suecica* (PCC 305), the diatom *Thalassiosira weissflogii* (CCAP 1085/1) and the dinoflagellate *Protoceratium reticulatum* (PRA 0206) were grown axenically in 150 mL glass tubes (19 cm x 3.5 cm) filled with 100 mL of ESAW (Enriched Seawater Artificial Medium; [8]) buffered with 10 mM Tris-HCl, pH 8.0. Each species was cultured in the presence of 1 mM, 5 mM, 10 mM or 30 mM Na_2_SO_4_ in order to mimic changes in marine [SO_4_^2-^] from the Proterozoic to the present [5]. The osmolarity of the medium was kept constant at 0.45 mol L^-1^ using NaCl. Cultures were maintained at 20°C under a continuous photon flux density (PFD) of 120 μmol photons m^-2^ s^-1^, provided by cool white fluorescent tubes. All experiments were carried out on cells in the exponential growth phase allowed to grow at the given SO_4_^2-^ concentration for at least 4 generations prior to any measurement. 

For the second experiment, designed to assess the impact of protistan grazers on the response to changes in sulphate concentrations, the ciliate *Euplotes* sp. was added to monospecific algal cultures in a proportion of about 1 ciliate cell per 0.05-0.1 μg of algal C [5]. Growth conditions for these cultures were otherwise the same as for the first experiment. All experiments were carried out on cells in the exponential growth phase allowed to grow at the given SO_4_^2-^ concentration in the presence of *Euplotes* sp. for at least 4 generations prior to any measurement. 

For the third set of experiments, designed to assess the impact of small metazoan grazers on the response to changes in sulphate concentrations, the calanoid copepod *Acartia tonsa* was added to monospecific algal cultures in a proportion of about 1 copepod per 0.2-0.7 mg of algal C. This range was used to have a saturating food level for copepod [9–11] and to maintain a ratio between animal and algal C similar to the previous experiment with *Euplotes* sp. (200 algal C to 1 animal C). Other culture parameters were the same as for the first experiment. 

The natural mortality of the copepods was estimated by preliminary tests: accordingly new copepods were added to the algae culture at every dilution, in order to assure a constant ratio between grazers and algal cells. In the case of *T. weissflogii*, however, the experiments were conducted on the cells collected after 10 days since the copepods were added. This time was selected because it allowed the acclimation of algae to the presence of grazers but also because after a longer period the copepods started dying within 24 hours after they were added to the cultures. In the case of *Synechococcus* sp. the copepods died within 24 hours after addition to the culture, presumably from cyanobacterial toxins. For this reason, the copepods were replaced every day; this method allowed the evaluation of algal cell composition, but did not permit a reliable evaluation of the specific growth rate.

The grazers and their visible wastes (e.g. copepods fecal pellets) were separated from the culture by filtration prior to any measurement. In order to get rid of any residual wastes, further washes were effected with fresh medium and an ammonium formate solution isosmotic to the culture media. The impact of wastes that may have remained in the culture was also tested in preliminary checks and found to be negligible in all cases and for all measurements.

### Growth rate, cell size and dry weight, chlorophyll fluorescence

Cell numbers and volumes were measured with automatic cell counter CASY TT (Innovatis AG, Retlingen, Germany; [12]). Specific growth rates, μ, were derived from daily counts of exponentially growing cells, carried out on a minimum of three distinct cultures for each treatment. In the experiments conducted in the presence of ciliates or copepods, grazer feeding rate was not considered; the growth rates are thus net of grazing. For dry weight determination, cells were washed with an isosmotic ammonium formate solution and dried at 100°C until weight stabilized. Measurements were conducted for at least three independent cultures.

The chlorophyll fluorescence associated to PSII was studied using a Dual-PAM-100 fluorometer according to [5]. The maximum fluorescence yield and the dark fluorescence yield of dark adapted (Fm and Fo, respectively) and illuminated (Fm’ and Fo’) cells were determined. The maximal quantum yield of PSII and the quenching parameters qP and NPQ were derived from these measurements according to [13,14]. Data acquisition and analysis were conducted using the Dual-PAM v1.8 software (Walz GmbH, Effeltrich, Germany).

### Elemental composition

Cell quotas of C, N and S were determined using an elemental analyzer (EA1108, Carlo Erba Instruments, Milan, Italy) as described in [5]. One to six milligrams of cells (dry weight) were washed twice with an ammonium formate solution isosmotic to the culturing media and dried at 80°C until the weight stabilized. The washes with ammonium formate (which is volatile at high temperature) were necessary to eliminate growth medium salts that would have interfered with the determination of dry weight and, consequently, cell stoichiometry. Sulphanilamide (C:N:S= 6:2:1) was used as a standard. Elemental quotas were calculated as pico- or femto-grams per cell, normalized to cell dry weight and to cell volume. Data acquisition and analysis were performed with the software EAS-Clarity (DataApex Ltd. 2006, Czech Republic). All measurements were repeated for four independent cultures. 

The abundance of elements with an atomic mass between 24.305 g mol^-1^ (Mg) and 238.03 g mol^-1^ (U) was measured using a Total Reflectance X-ray Fluorescence spectrometer (S2 Picofox, Bruker AXS Microanalysis GmbH, Berlin Germany). Dry algae cultured at 5 mM or 30 mM SO_4_^2-^ (10^10^-10^11^ cells L^-1^) were resuspended in 1 mL of dH_2_O and vortexed until the suspension was homogeneous. A solution of 1 g L^-1^ Ga (Sigma Aldrich, St. Luis, MO, USA) in 5% HNO_3_ was added as internal standard to a final concentration of 5 μL L^-1^. Aliquots of 10 µL of this suspension were deposed on a quartz sample holder, dried on a heating plate for 10 minutes, and measured for 1000 seconds. Spectral deconvolution and quantification of elemental abundances were performed by the SPECTRA 6.1 software (Bruker AXS Microanalysis GmbH, Berlin, Germany). 

### Organic composition

Cells for protein determination were harvested by centrifugation. For each replicate, a volume of culture containing 2 to 10·10^6^ cells was used. The determination was conducted according to [15].

Samples for FTIR analysis were prepared as described by [12,16]. FTIR spectra were acquired with a Tensor 27 FTIR spectrometer (Bruker Optics, Ettlingen, Germany). Bands were attributed to cellular pools according to [17].

Relative ratios of carbohydrates, lipids, proteins, and silica were calculated from the band integrals, using the OPUS 6.5 software (Bruker Optik GmbH, Ettlingen, Germany). The calculation of the band integrals assigned to lipids, proteins and carbohydrates was performed using the “Integral B Function” of the software. For the diatom *T. weissflogii* the evaluation of the carbohydrate pool from FTIR spectra was hampered by the fact that some of the typical carbohydrates bands were masked by the silica features (1075 cm^-1^; [18]). Because of that the carbohydrate contribution was not considered for this species.

Semi-quantification of carbohydrate, lipid and silica pools was performed according to protocols in [12]. The abundance of carbohydrates, lipids and silica in a given cell type was expressed relative to their abundance in a cell type used as reference, the reference being cells cultured in the presence of the modern SO_4_^2-^ concentration and in the absence of grazers.

The overall degree of reduction of cell organic constituents was derived from the ratio between the sum of the absorbance of CH_2_- and CH_3_- and that of CH-, according to [12]. Since the numerical value of this ratio is not directly equivalent to the absolute level of reduction, but rather indicates the change in the relative proportion of the different -CH_n_ groups, it was termed ‘reduction index.’ 

### Allelopathic activity of *Synechococcus* sp. and *T. weissflogii*

The observations that copepods started dying in cultures of *T. weissflogii* grown in the presence of *A. tonsa* for 20 days, and that copepods died within 24 hours in the cultures with *Synechococcus* sp. suggested the presence of anti-grazing compounds. To investigate this possibility further, allelopathic tests were carried out. The mortality of copepods was tested using spent media of the cultures after filtration of algal cells and using *T. suecica* as “safe” food. 

For *T. weissflogii* we tested:

1. Spent medium from *T. weissflogii* culture (5 mM, 30 mM SO_4_^2-^)
2. Spent medium from *T. weissflogii* + *A. tonsa* 10 days old culture (5 mM, 30 mM SO_4_^2-^)
3. Spent medium from *T. weissflogii* + *A. tonsa* 20 days old culture (5 mM, 30 mM SO_4_^2-^)

For *Synechococcus* sp. we tested:

1. Spent medium from *Synechococcus* sp. cultures (5 mM, 30 mM SO_4_^2-^)
2. Spent medium from *Synechococcus* + *A. tonsa* culture (5 mM, 30 mM SO_4_^2-^)

### Statistics

Data are reported as mean ± standard deviation for measurements obtained from at least three distinct cultures. Statistical significance of differences among the means was determined by analysis of variance (ANOVA) and Tukey’s post-hoc test, using GraphPad Prism 4.03 software (GraphPad Software, San Diego, CA, USA), with the level of significance set at 95%.

## Results

In general, our experimental results support the motivating hypothesis: the addition of grazers influenced both the growth rates and biochemical composition of phytoplankton species and did so in a taxon-specific way. There, was, however, only limited evidence for an interactive effect of grazing and sulphate abundance.

### Growth rate, cell size, dry weight and chlorophyll fluorescence

**Figure 1:**
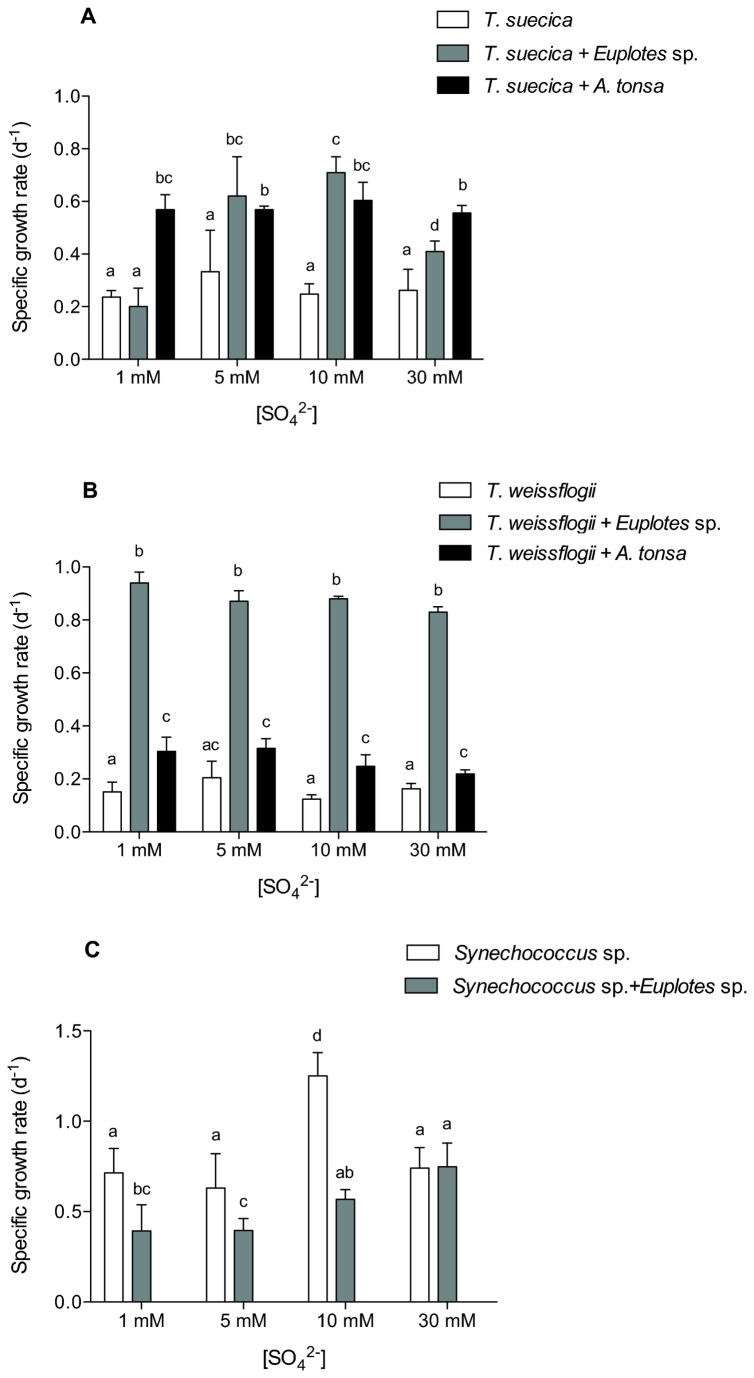
Effect of grazers on the specific growth rate of cultured algae. Specific growth rate of (A) *T. suecica*, (B) *T. weissflogii* and (C) *Synechococcus* sp. cultured at 1 mM, 5 mM, 10 mM or 30 mM SO_4_^2-^, and in the presence of the ciliate *Euplotes* sp. or the copepod *Acartia tonsa*. Error bars represent standard deviation calculated from at least three independent replicates. Letters above the histograms indicate statistical significance of differences: when the same letter appears on top of more than one bar, those values are not significantly different; different letters identify statistically different means (p > 0.05).

The dinoflagellate *Protoceratium reticulatum* encysted in the presence of either ciliate (*Euplotes* sp.) or copepod (*Acartia tonsa*) grazers and so could not be monitored for growth rate. For our other experimental phytoplankton cultures, however, grazers had a significant effect on net specific growth rate – positive in the eukaryotic taxa and negative in the cyanobacterial culture. The specific growth rate of the green alga *Tetraselmis suecica* increased and did so regardless of grazer type (p < 0.05; Figure 1A). Only at 1 mM SO_4_^2-^ was there no obvious effect of ciliate grazing on the growth rate of the green alga. The specific growth rate of the diatom *Thalassiosira weissflogii* was also higher in the presence of the two grazers; in this alga, growth rate was 3-fold higher when cells were cultured in the presence of ciliates than when they grew in the presence of the copepod (2-fold stimulation of growth; p < 0.05; Figure 1B). At most sulphate levels, *Synechococcus* sp. (Figure 1C) also responded to the presence of ciliate grazers, but, contrary to the eukaryotic algae, showed a decrease in net growth rates. The copepods did not persist in the cyanobacterial culture for more than one day (See below). For this reason, copepods were added daily to evaluate the effect of the grazer on cell composition, but this procedure did not allow a reliable determination of the net growth rate, due to the unknown impact of the toxin on copepod grazing. 

For *T. suecica*, cell volume and dry weight were not affected by the presence of either grazer (p > 0.05; Tab. 1). In contrast, the diatom exhibited a marked decrease in both cell volume and dry weight when grown in the presence of copepods (p < 0.05; Tab. 1). Cells of *Synechococcus* sp. cultured in the presence of *Euplotes* sp. showed a higher cellular volume and a lower dry weight than cells grown in the absence of grazers. *Synechococcus* cells were both larger and doubled their dry weight when cultured in the presence of *Acartia* (p < 0.05; Tab. 1).

**Table 1:** Functional responses of microalgae to the presence of grazers.

Species	[SO_4_^2-^]	Cell volume	Dry weightnbsp;	Fv/Fm	NPQ	qP
	*mM*	*μm^3^*	*pg cell^-1^*			
*T. suecica*	1	446.0 ± 192.8	109.2 ± 31.39	0.62 ± 0.06	0.32 ± 0.27	0.79 ± 0.22
*T. suecica*	5	466.5 ± 178.6	129.3 ± 68.94	0.62 ± 0.05	0.67 ± 0.22	0.72 ± 0.09
*T. suecica*	10	484.8 ± 202.1	107.9 ± 37.29	0.64 ± 0.06	0.52 ± 0.27	0.75 ± 0.12
*T. suecica*	30	387.5 ± 139.6	121.4 ± 25.22	0.63 ± 0.02	0.47 ± 0.38	0.75 ± 0.10
*T. suecica* + *Euplotes* sp.	1	288.5 ± 54.82	118.9 ± 78.54	0.61 ± 0.05	0.30 ± 0.18	0.79 ± 0.07
*T. suecica* + *Euplotes* sp	5	274.5 ± 42.67	197.6 ± 88.79	0.63 ± 0.03	0.24 ± 0.15	0.78 ± 0.09
*T. suecica* + *Euplotes* sp	10	282.3 ± 53.52	175.1 ± 17.21	0.63 ± 0.04	0.27 ± 0.17	0.81 ± 0.07
*T. suecica* + *Euplotes* sp	30	297.7 ±57.55	152.5 ± 57.14	0.59 ± 0.02	0.31 ± 0.30	0.84 ± 0.09
*T. suecica* + *A. tonsa*	1	336.0 ± 19.53	94.38 ± 13.94	0.65 ± 0.04	0.19 ± 0.04	0.77 ± 0.02
*T. suecica* + *A. tonsa*	5	348.0 ± 14.72	108.6 ± 24.30	0.67 ± 0.01	0.25 ± 0.17	0.78 ± 0.08
*T. suecica* + *A. tonsa*	10	329.3 ± 22.46	100.9 ± 15.95	0.68 ± 0.04	0.18 ± 0.02	0.80 ± 0.04
*T. suecica* + *A. tonsa*	30	341.3 ± 27.68	96.32 ±15.18	0.69 ± 0.03	0.16 ± 0.05	0.81 ± 0.06
*T. weissflogii*	1	972.1 ± 296.6	390.6 ± 39.47	0.46 ± 0.09	0.40 ± 0.36	0.87 ± 0.09
*T. weissflogii*	5	1098 ± 363.9	421.3 ± 176.1	0.48 ± 0.04	0.42 ± 0.25	0.88 ± 0.03
*T. weissflogii*	10	1065 ± 374.7	295.2 ±189.7	0.47 ± 0.12	0.51 ± 0.46	0.89 ± 0.04
*T. weissflogii*	30	1087 ± 350.3	424.0 ±113.5	0.54 ± 0.08	0.53 ± 0.66	0.79 ± 0.02
*T. weissflogii* + *Euplotes* sp.	1	1100 ± 396.7	247.1 ± 134.2	0.55 ± 0.06	0.54 ± 0.29	0.90 ± 0.05
*T. weissflogii* + *Euplotes* sp.	5	1071 ± 405.3	347.5 ±191.2	0.55 ± 0.08	0.33 ± 0.26	0.89 ± 0.04
*T. weissflogii* + *Euplotes* sp.	10	1200 ± 402.2	304.9 ±124.2	0.55 ± 0.04	0.47 ± 0.38	0.93 ± 0.02
*T. weissflogii* + *Euplotes* sp.	30	1294 ± 381.3	519.3 ± 369.2	0.56 ± 0.07	0.44 ± 0.37	0.90 ± 0.03
*T. weissflogii* + *A. tonsa*	1	504.5 ± 51.61	113.3 ± 34.98	0.45 ± 0.07	0.24 ± 0.18	0.69 ± 0.21
*T. weissflogii* + *A. tonsa*	5	523.2 ± 39.73	151.8 ± 25.97	0.52 ± 0.04	0.25 ± 0.13	0.79 ± 0.19
*T. weissflogii* + *A. tonsa*	10	482.2 ± 25.98	120.3 ± 31.81	0.54 ± 0.05	0.22 ± 0.18	0.78 ± 0.17
*T. weissflogii* + *A. tonsa*	30	480.2 ± 24.48	112.6 ± 21.68	0.54 ± 0.04	0.15 ± 0.10	0.80 ± 0.15
*Synechococcus* sp.	1	9.70 ± 0.80	2.36 ± 0.49	0.13 ± 0.00	0.01 ± 0.01	0.65 ± 0.27
*Synechococcus* sp.	5	9.32 ±0.60	1.95 ± 0.34	0.16 ± 0.03	0.01 ± 0.01	0.68 ± 0.09
*Synechococcus* sp.	10	10.1 ± 1.35	2.41 ± 0.64	0.13 ± 0.02	0.00 ± 0.00	0.69 ± 0.14
*Synechococcus* sp.	30	9.70 ± 1.26	2.61 ± 0.74	0.14 ± 0.03	0.02 ± 0.03	0.59 ± 0.16
*Synechococcus* sp. + *Euplotes* sp.	1	13.2 ± 2.81	1.57 ± 0.30	0.29 ± 0.05	0.08 ± 0.01	0.40 ± 0.09
*Synechococcus* sp. + *Euplotes* sp.	5	12.3 ± 1.56	1.55 ± 0.48	0.23 ± 0.07	0.06 ± 0.04	0.43 ± 0.14
*Synechococcus* sp. + *Euplotes* sp.	10	12.5 ± 0.59	2.19 ± 0.47	0.26 ± 0.14	0.04 ± 0.04	0.59 ± 0.36
*Synechococcus* sp. + *Euplotes* sp.	30	10.2 ± 0.90	2.10 ± 0.06	0.23 ± 0.08	0.02 ± 0.01	0.54 ± 0.23
*Synechococcus* sp. + *A. tonsa*	1	13.6 ± 2.23	4.42 ± 0.99	n.d.	n.d.	n.d.
*Synechococcus* sp. + *A. tonsa*	5	15.9 ± 0.45	4.35 ± 0.74	n.d.	n.d.	n.d.
*Synechococcus* sp. + *A. tonsa*	10	14.3 ± 0.28	2.95 ± 1.00	n.d.	n.d.	n.d.
*Synechococcus* sp. + *A. tonsa*	30	13.3 ±1.31	2.69 ± 0.71	n.d.	n.d.	n.d.

Cell volume, cell dry weight, maximal PSII quantum yield (Fv/Fm), non photochemical quenching coefficient (NPQ) and photochemical quenching coefficient (qP) are shown for *Tetraselmis suecica*, *Thalassiosira weissflogii*, *Synechococcus* sp. cultured at 1, 5, 10 or 30 mM SO_4_^2-^, in the presence or absence of *Euplotes* sp. or *Acartia tonsa*. Results are shown as means ± standard deviations calculated from at least 4 independent replicates.

n.d.: not determined (see text for details)

The maximal PSII quantum yield (Fv/Fm) of all algal species was not affected by the presence of grazers during growth (p > 0.05; Tab. 1). In both *T. suecica* and *T. weissflogii*, regardless of [SO_4_^2-^], the presence of grazers resulted in a lower non-photochemical quenching (NPQ; p < 0.05; Tab. 1). Instead, *Synechococcus* sp. cells acclimated to 1 mM SO_4_^2-^ and to the presence of ciliates showed an order of magnitude higher NPQ than cells cultured in the absence of grazers. The grazer effect decreased with increasing sulphate availability and disappeared completely at the highest sulphate concentrations (p < 0.05; Tab. 1). The photochemical quenching coefficient (qP) did not change in the green alga and in the diatom, as a function of [SO_4_^2-^] or of the presence of grazers (p > 0.05; Tab. 1). *Synechococcus* sp. cells had a lower qP coefficient at all [SO_4_^2-^] when *Euplotes* sp. was present than when cultured without grazers (p < 0.05; Tab. 1).

### Elemental and organic composition

In *T. suecica*, the relative abundance of C, N, and P did not vary systematically as a function of either [SO_4_^2-^] or grazer presence (Tab. 2). The diatom, *T. weissflogii* generally showed lower C:P and N:P than *T. suecica* and, especially, had lower C:P and N:P in the presence of the copepod grazer. *Synechococcus* sp. generally had lower C:P and N:P than the eukaryotic algae, and both ratios were higher in the presence of grazers. Thus, in our experiments, both protistan and small metazoan grazers influenced C:N:P stoichiometry, but in a species-specific way not easily generalized. Normalization to S again shows no general trend with respect to treatment for *T. suecica*, but an increase in S:C for *T. weissflogii*.

**Table 2:** C, N and S mass ratio relative to P in *Tetraselmis suecica*, *Thalassiosira weissflogii* and *Synechococcus* sp. cells grown in the presence of either protist (*Euplotes* sp.) or microarthropode (*Acartia tonsa*) grazers, at 5 or 30 mM SO_4_^2-^.

Species	[SO42-]	Element mass ratio				P content	Dry weight
	*mM*	C	N	S	P	*fg cell^-1^*	*pg cell^-1^*
*T. suecica*	5	125 ± 7.39	22.7 ± 3.54	2.00 ± 1.09	1.00 ± 0.55	446 ± 244	129 ± 68.9
*T. suecica*	30	104 ± 6.11	18.9 ± 1.85	1.46 ± 0.30	1.00 ± 0.21	503 ± 105	121 ± 25.2
*T. suecica* + *Euplotes* sp.	5	209 ± 34.8	35.3 ± 2.12	4.00 ± 1.18	1.00 ± 0.58	490 ± 285	198 ± 88.8
*T. suecica* + *Euplotes* sp.	30	88.1 ± 9.35	12.9 ± 3.82	1.95 ± 1.47	1.00 ± 0.85	753 ± 416	152. ± 57.1
*T. suecica* + *A. tonsa*	5	110 ± 7.44	20.6 ± 1.56	1.39 ± 0.21	1.00 ± 0.12	425 ± 52.6	109 ± 24.3
*T. suecica* + *A. tonsa*	30	110 ± 4.78	20.8 ± 1.38	1.35 ± 0.41	1.00 ± 0.14	385 ± 53.3	96.3 ± 15.8
*T. weissflogii*	5	81.7 ± 6.13	11.5 ± 1.19	1.28 ± 0.54	1.00 ± 0.42	2014 ± 844	421 ± 176
*T. weissflogii*	30	140 ± 5.40	20.6 ± 1.80	2.63 ± 0.71	1.00 ± 0.27	1101 ± 300	424 ±113
*T. weissflogii* + *Euplotes* sp.	5	97.8 ± 13.0	16.0 ± 0.84	1.93 ± 0.00	1.00 ± 0.00	1300 ± 492	347 ±191
*T. weissflogii* + *Euplotes* sp.	30	103 ± 5.50	15.8 ± 0.65	1.86 ± 0.00	1.00 ± 0.00	1895 ± 0.00	519 ± 369
*T. weissflogii* + *A. tonsa*	5	48.1 ± 3.54	6.98 ± 0.77	1.22 ± 0.31	1.00 ± 0.36	1413 ± 508	152 ± 25.9
*T. weissflogii* + *A. tonsa*	30	27.7 ± 2.79	4.46 ± 0.21	0.83 ± 0.07	1.00 ± 0.45	1640 ± 734	113 ± 21.7
*Synechococcus* sp.	5	20.6 ± 0.95	3.86 ± 0.46	0.23 ± 0.04	1.00 ± 0.17	37.4 ± 6.43	1.95 ± 0.34
*Synechococcus* sp.	30	20.1 ± 0.75	3.86 ± 0.32	0.27 ± 0.08	1.00 ± 0.28	51.9 ± 14.8	2.61 ± 0.74
*Synechococcus* sp. + *Euplotes* sp.	5	508 ± 20.4	112 ± 5.32	0.27 ± 0.08	1.00 ± 0.29	2.59 ± 1.51	1.55 ± 0.48
*Synechococcus* sp. + *Euplotes* sp.	30	53.4 ± 4.85	10.8 ± 0.78	0.61 ± 0.07	1.00 ± 0.67	16.2 ± 10.8	2.10 ± 0.06
*Synechococcus* sp. + *A. tonsa*	5	68.1 ± 5.95	10.1 ± 0.95	0.47 ± 0.13	1.00 ± 0.34	25.9 ± 8.90	4.35 ± 0.74
*Synechococcus* sp. + *A. tonsa*	30	14.6 ± 0.32	2.52 ± 0.65	0.30 ± 0.09	1.00 ± 0.60	76.6 ± 45.9	2.69 ± 0.71

Results are shown as means ± standard deviations calculated for at least 4 independent replicates.

Minor elements vary among species and with treatment, although the diatom *T. weissflogii*, showed greater homeostasis with respect to elemental composition than the green alga or the cyanobacterium. Trace element abundances for each experimental treatment can be found in supplemental information. In general, there is no obvious trend in these data (Tab. S1). 

Interestingly, the experimental treatments show more systematic variations with respect to organic composition than elemental stoichiometry. In *T. suecica*, the lipid to protein, carbohydrate to protein and carbohydrate to lipid ratios, as obtained with Fourier Transform InfraRed spectroscopy (FTIR), were higher when *Euplotes* sp. was added to the cultures (p < 0.05; Figure S8, S9, S10); the carbohydrate to protein and the carbohydrate to lipid ratios were even higher in cells acclimated to 1 mM, 5 mM and 10 mM SO_4_^2-^ when *A. tonsa* was present (p < 0.05; Figure S8, S9, S10). *T. weissflogii* cells acclimated to growth at 1 mM SO_4_^2-^ and to the presence of grazers had a significantly higher lipid to protein ratio (~ 3.5 times, p < 0.05; Figure S8.); in cells acclimated to higher SO_4_^2-^, this ratio was two times higher when grazers were present (p < 0.05; Figure S1F). The protein to silica ratio was lower in the presence of copepods (p < 0.05; Figure S11).

*Synechococcus* sp. cells acclimated to 1 mM or 5 mM SO_4_^2-^ had a higher lipid to protein ratio when cultured in the presence of grazers (~6 times higher with *Euplotes* sp.; ~30 times with *A. tonsa*; p < 0.05 Figure S8). At higher [SO_4_^2-^], this ratio was 6-fold higher when the algae grew with the grazers, irrespective of the grazer type (p < 0.05; Figure S8). The carbohydrate to protein ratio of cells acclimated to 1 mM or 5 mM [SO_4_^2-^] was significantly lower when the ciliates and the copepod were present in the cultures (p < 0.05; Figure S9). Grazers resulted in a significant decrease (~10 times) of the carbohydrate to lipid ratio in *Synechococcus* sp. cells regardless of growth [SO_4_^2-^] (p < 0.05; Figure S10).

**Figure 2:**
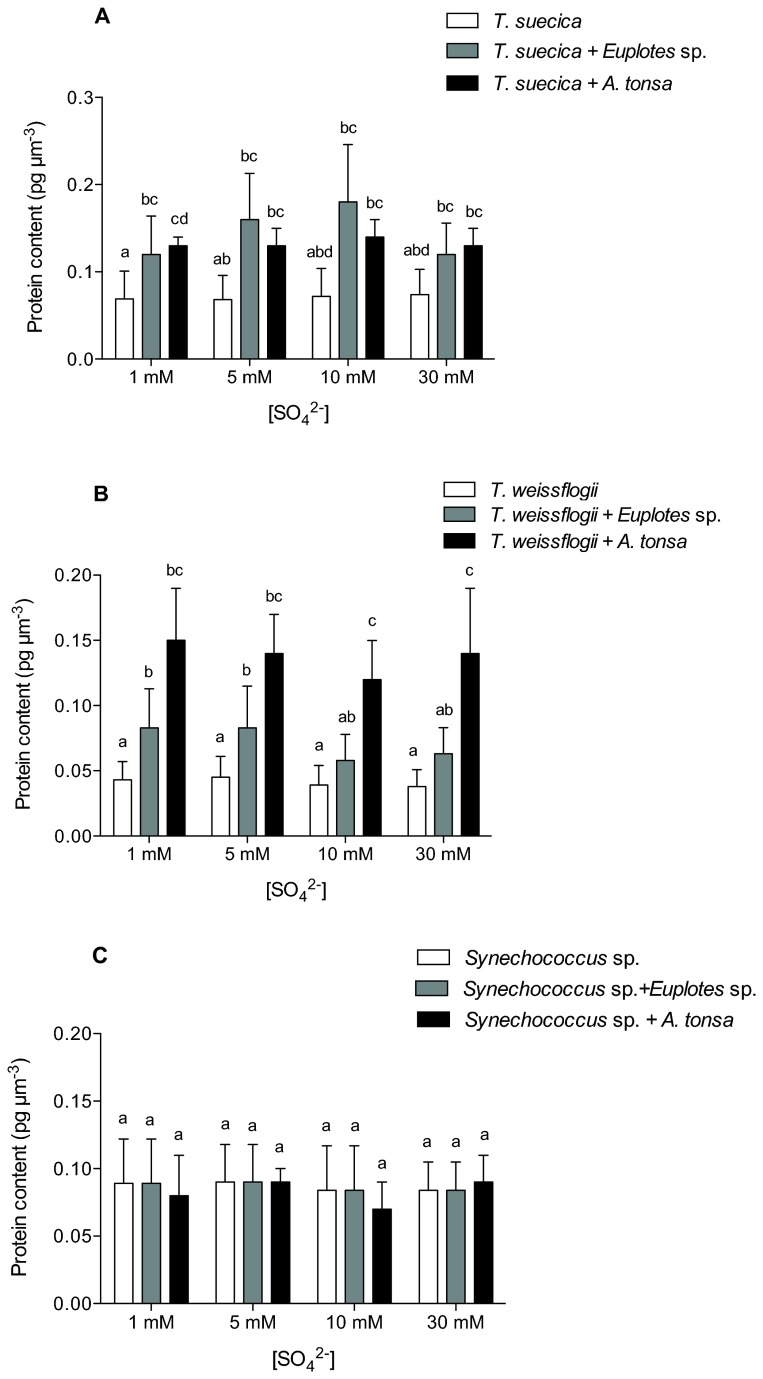
Effect of grazers on algal protein content. Protein content of (A) *T. suecica*, (B) *T. weissfloggi*, and (C) *Synechococcus* sp. cells cultured at 1 mM, 5 mM, 10 mM or 30 mM SO_4_^2-^ and in the presence of *Euplotes* sp. or *A. tonsa*. Error bars represent standard deviation calculated for at least four independent replicates. Histograms on top of which the same letter appears represent means that are not statistically different; different letters identify means that are significantly different (p > 0.05).

The absolute amount of protein normalized per cell volume was significantly higher in cells of *T. suecica* and *T. weissflogii* acclimated to the presence of grazers than in cells cultured in their absence, regardless of the sulphate concentration in the growth medium (p < 0.05; Figure 2A, B). In *T. weissflogii*, an effect of the type of grazer was observed: the amount of protein per volume unit was significantly higher in cells cultured in the presence of *A. tonsa* than in cells grown with *Euplotes* sp. (p < 0.05; Figure 2B). No variation in protein content was found in *Synechococcus* sp. cells (p > 0.05; Figure 2C).

**Figure 3:**
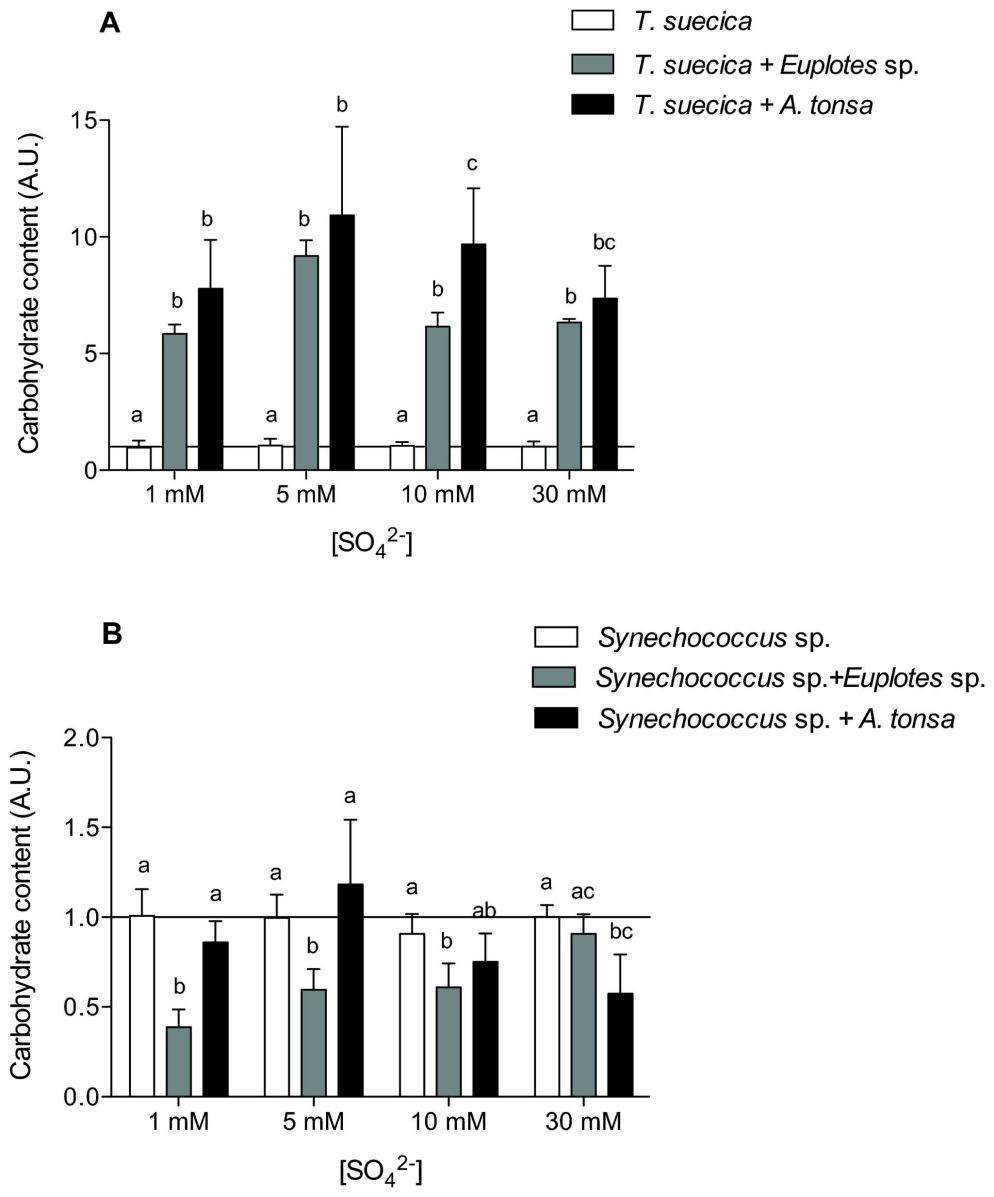
Effect of grazers on algal carbohydrate content. Relative amount of carbohydrate of (A) *T. suecica* and (B) *Synechococcus* sp. cells cultured in the presence of 1 mM, 5 mM, 10 mM or 30 mM SO_4_^2-^ and of *Euplotes* sp. or *A. tonsa*. Carbohydrates were estimated from FTIR absorbances according to Palmucci et al. (2011), normalized for cellular volume and relative to to values obtained from cells cultured at 30 mM SO_4_^2-^ and in the absence of grazers. Error bars represent standard deviation calculated for at least four independent replicates. When the same letter appears above more than one histogram, those values are not statistically different; different letters identify means that are significantly different (p < 005).

The carbohydrate and the lipid contents were estimated applying the equations proposed by [12], using the FTIR data and the absolute cell protein content. The carbohydrate content in *T. suecica* was strongly affected by the presence of grazers; the cells acclimated to the presence of *Euplotes* sp. showed a carbohydrate content expressed per unit of cell volume that was 5-7 times higher than that of cells cultured in its absence (p < 0.05; Figure 3A). When *A. tonsa* was present at high sulphate concentration, the carbohydrate content was even higher (~7-10-fold that of control cultures; p < 0.05; Figure 3A). *Synechococcus* sp., when cultured in the presence of *A. tonsa*, had similar carbohydrate content in the presence and absence of grazers (p > 0.05; Figure 3B); when the cyanobacterium was grown with *Euplotes* sp., the amount of carbohydrate per unit of cell volume was usually *lower* than that of cells grown in the absence of grazers (p < 0.05; Figure 3B). 

**Figure 4:**
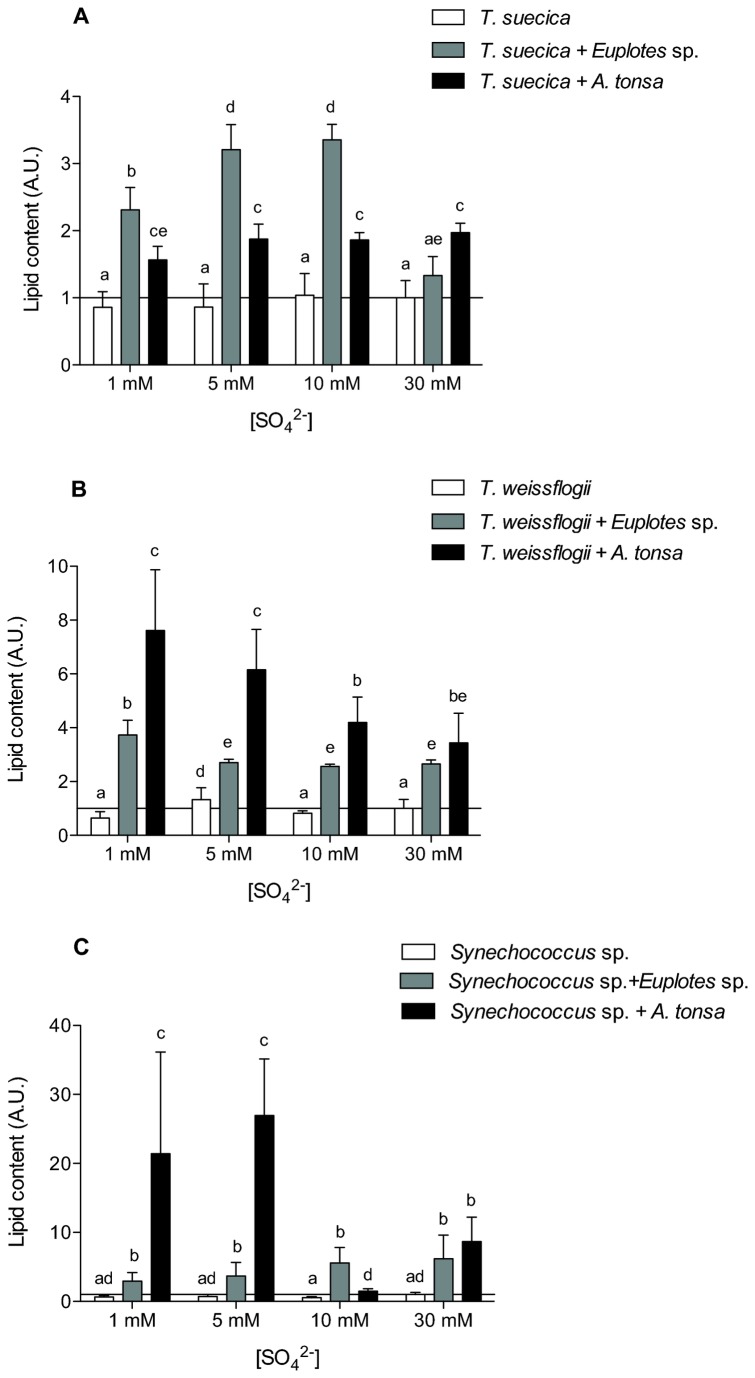
Effect of grazers on algal lipid content. Relative amount of lipid of (A) *T. suecica*, (B) *T. weissflogii* and (C) *Synechococcus* sp. cells cultured in the presence of 1 mM, 5 mM, 10 mM or 30 mM SO_4_^2-^ and of *Euplotes* sp. or *A. tonsa*. Lipids were estimated from FTIR absorbances according to Palmucci et al. (2011), normalized for cellular volume and relative to values obtained from cells cultured at 30 mM SO_4_^2-^ and in the absence of grazers. Error bars represent standard deviation calculated for at least four independent replicates. Histograms on top of which the same letter appears represent means that are not statistically different; different letters identify means that are significantly different (p > 0.05).

In all three algal species, the presence of grazers resulted in a higher amount of lipid per unit of cell volume (p < 0.05; Figure 4A, B, C). This was especially obvious in *Synechococcus* sp. acclimated to *A. tonsa* at 1 mM and 5 mM SO_4_^2-^ (p < 0.05; Figure 4C). In *T. weissflogii*, in *Synechococcus* sp. (except the cells cultured at 30 mM SO_4_^2-^), and in *T. suecica* at 30 mM SO_4_^2-^, the presence of *A. tonsa* caused a larger increase of the lipid pool than that of *Euplotes* sp. (p < 0.05; Figure 4A, B, C). 

**Figure 5:**
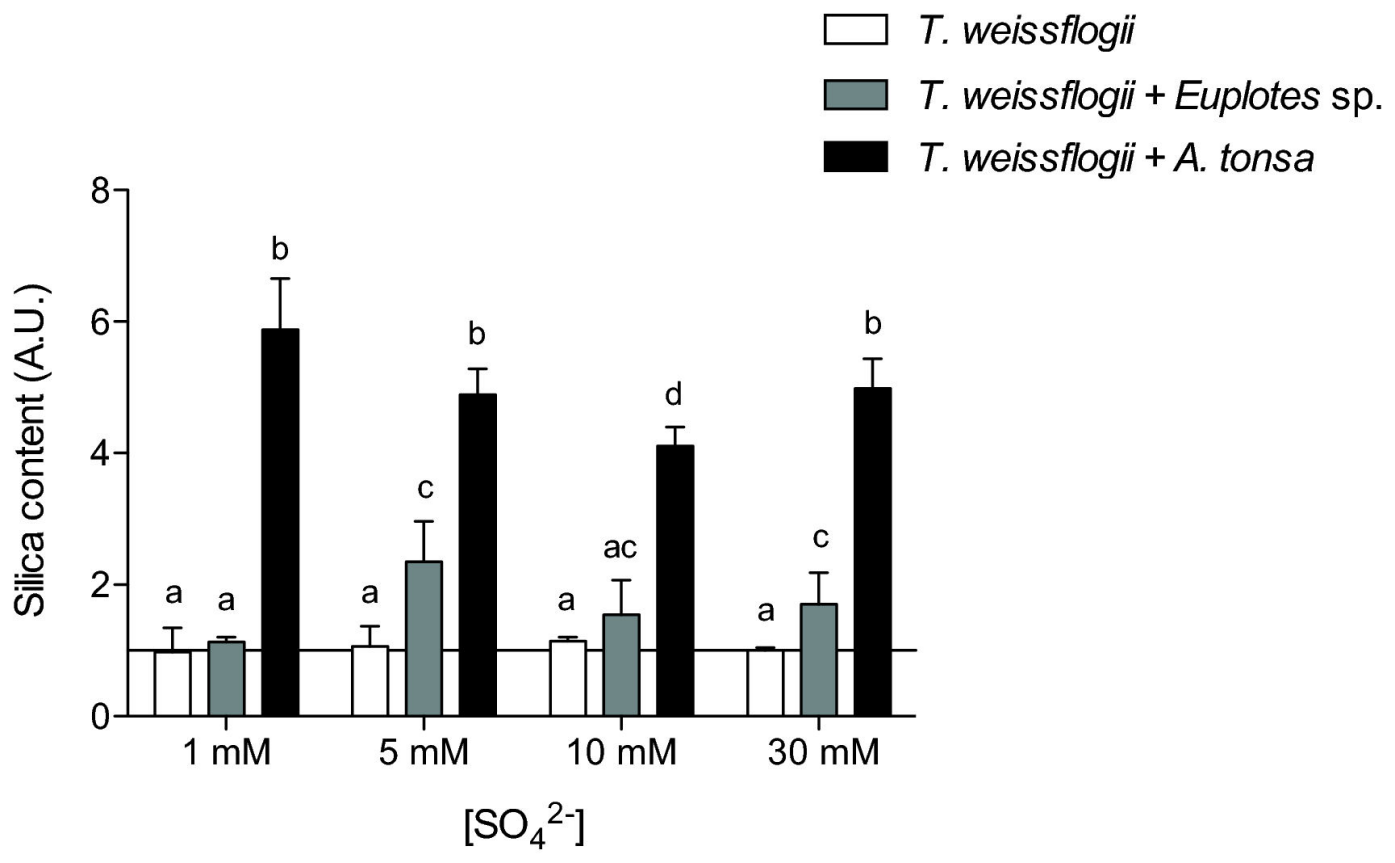
Effect of grazers on the amount of silica in *Thalassiosira weissflogii* cells. This figure depicts the relative amount of silica in *T. weissflogii* cells cultured at 1, 5, 10 or 30 mM SO_4_^2-^ and in the presence of either *Euplotes* sp. or *Acartia tonsa*. Silicate was estimated from FTIR absorbances according to Palmucci et al. (2011), normalized for cell volume and relative to values obtained from cells cultured at 30 mM SO_4_^2-^ and in the absence of grazers. Error bars represent standard deviation calculated from at least four independent replicates. Histograms on top of which the same letter appears represent means that are not statistically different; different letters identify means that are significantly different (p > 0.05).

In all treatments except at 1mM [SO_4_^2-^], the presence of ciliates resulted in a modest increase in silica. However, when the diatom *Thalassiosira weissflogii* grew in the presence of copepods, its silica content was a five- to six-fold higher. This occurred despite a concomitant decrease in cell size (p < 0.05; Figure 5) and it is thus suggestive of a greater thickness of the frustule.

**Figure 6:**
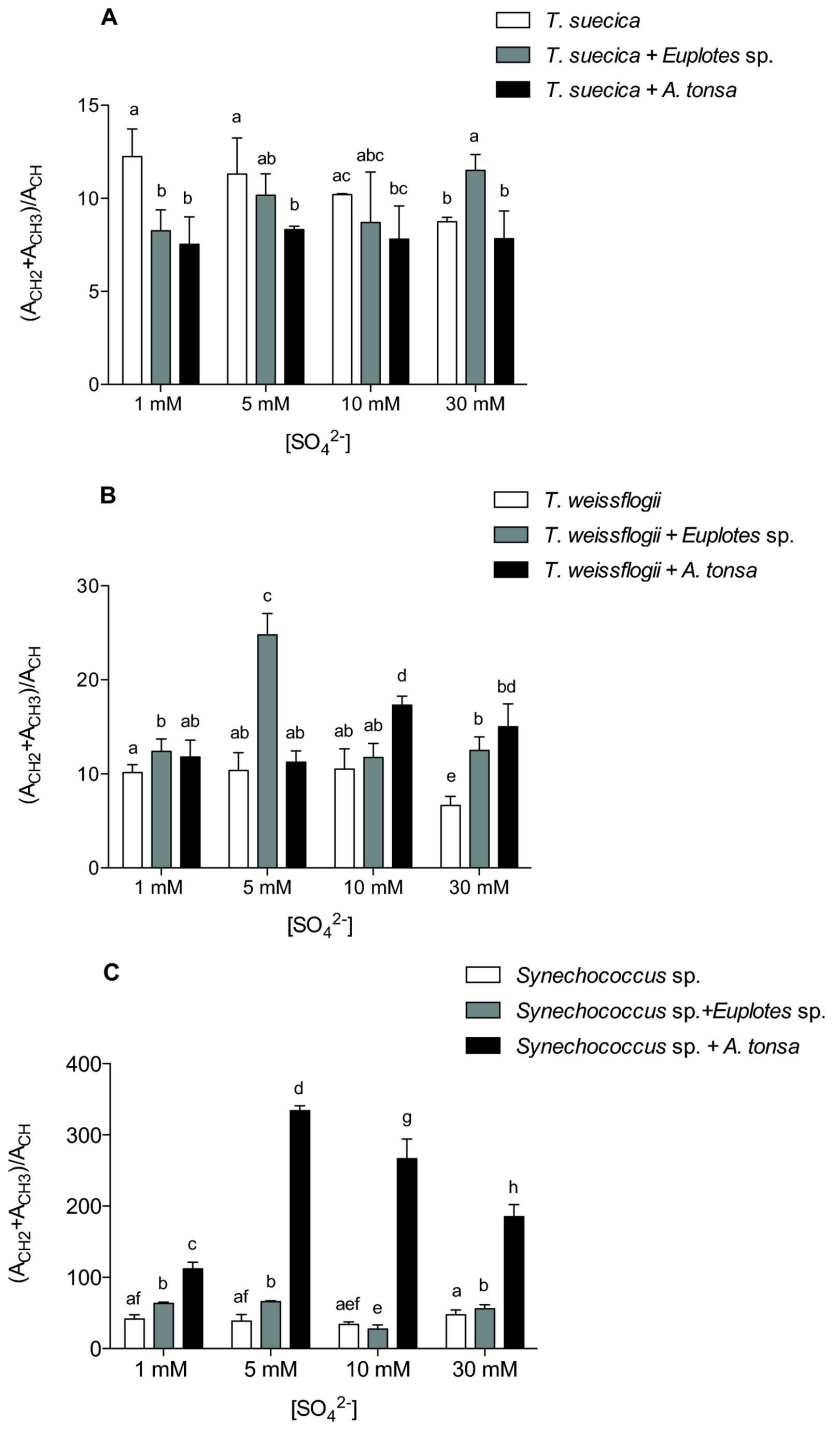
Effect of grazers on the level of reduction of organic cell constituents. This figure shows the overall level of reduction of organic constituents in (A) *Tetraselmis suecica*, (B) *Thalassiosira weissflogii* and (C) *Synechococcus* sp. cells cultured in media containing 1, 5, 10 or 30 mM SO_4_^2-^ and in the presence of either *Euplotes* sp. or *Acartia tonsa*. Error bars show standard deviation calculated from at least four independent replicates. Histograms on top of which the same letter appears represent means that are not statistically different; different letters identify means that are significantly different (p > 0.05).

Finally, the overall level of reduction of the cell organic constituents of *T. suecica* cells, as the ratio of the infrared absorbances of the (-CH_3_ + -CH_2_) and –CH groups, was appreciably lower when the algae were cultured in the presence of *A. tonsa*, except at 30 mM SO_4_^2-^ (p < 0.05; Figure 6A). The same effect was observed when *Euplotes* sp. was added to cells acclimated to 1 mM and 5 mM SO_4_^2-^, while at 30 mM the level of reduction of cells was higher (p < 0.05; Figure 6). In *T. weissflogii* and in *Synechococcus* sp. cells grazers typically had the opposite effect -- the reduction index of the algal organic matter was higher than in the cultures without grazers (p < 0.05; Figure 6B, C).

Interactive effects between sulphate abundance and grazing were limited, but not entirely absent. For example, for unknown reasons, *T. suecica* showed significant differences in growth rate (Figure 1A) and lipid content (Figure 3) responses to *Euplotes* at high [SO_4_^2-^]. These effects were not observed when *Acartia* was present or when grazers were absent. *Tetraselmis* also showed significant differences in lipid content when grown with *Euplotes* at varying sulphate levels. Elemental ratios showed a grazer x chemistry response as well, especially in *Synechococcus* cultures grown with *Euplotes*, with C:S and C:P (but not C:N) showing ten to twenty-fold differences between treatments with 5 and 30 mM [SO_4_^2-^] (Tab. 2). The reasons for these effects remain unclear.

### Allelopathic activity of *Synechococcus* sp. and *T. weissflogii*

When *Synechococcus* sp. was cultured in the presence of *A. tonsa*, but not of *Euplotes* sp., the entire copepod population was killed within 24 hours; in the case of *T. weissflogii*, all copepods died in about 20 days (Tab. 3). Thus, we checked the effect of the spent medium obtained from cultures of these algae that had been exposed to the grazers, and from pure algal cultures (Tab. 3). The results suggest that the presence of the copepod *A. tonsa* induced the production and the release into the external medium of anti-grazer compounds by *T. weissflogii* and *Synechococccus* sp., independent of [SO_4_^2-^] (Tab. 3). Copepod death was also observed in the presence of spent medium obtained from cultures of these algae, but not from media in which pure algal cultures were cultivated (Tab. 3). The toxic effect of such compounds was not detected for the green alga *T. suecica*, which therefore was used as “safe food” for copepods during the tests with spent media.

**Table 3:** Allelopathic activity.

	*T. weissflogii* + *A. tonsa* 10 days cultures			*T. weissflogii* + *A. tonsa* 20 days cultures			Spent medium from *T. weissflogii* pure culture			Spent medium from *T. weissflogii* + *A. tonsa* 10 days cultures			Spent medium from *T. weissflogii* + *A. tonsa* 20 days cultures			*Synechococcus* sp. + *A. tonsa* 1 day culture			Spent medium from *Synechococcus* sp.			Spent medium from *Synechococcus* sp. + *A. tonsa* 1 day culture	
	5 mM SO_4_^2^	30 mM SO_4_^2^		5 mM SO_4_^2^	30 mM SO_4_^2^		5 mM SO_4_^2-^	30 mM SO_4_^2-^		5 mM SO_4_^2-^	30 mM SO_4_^2-^		5 mM SO_4_^2-^	30 mM SO_4_^2-^		5 mM SO_4_^2^	30 mM SO_4_^2^		5 mM SO_4_^2-^	30 mM SO_4_^2-^		5 mM SO_4_^2-^	30 mM SO_4_^2-^	
*A. tonsa* viability	✓	✓		†	†		✓	✓		✓	✓		†	†		†	†		✓	✓		†	†

Copepod mortality was tested in the presence of either *Thalassiosira weissflogii* or *Synechococcus* sp., or using spent media of the algal cultures (with or without the grazers) after the algal cells were filtered out. In experiments with spent media, the copepod was fed with *T. suecica* as “safe food”. No effect was observed on the ciliates. These tests were carried out in triplicate. All replicates gave the same results in the same time.

✓ no effect on copepod viability; † the copepods died (see text for details).

## Discussion

Consistent with the motivating hypothesis, our results show phytoplankton responses to grazing that differ among taxa not only in magnitude but, commonly, in sign. Eukaryotic algae showed statistically significant increases in specific growth rate in the presence of grazers, whereas the cyanobacterium did not. In the presence of grazers, the eukaryotic phytoplankton also showed an increase in protein and carbohydrate content, whereas the cyanobacteria did not (Figures 2, 3). That is, unlike the cyanobacterium, which showed only modest changes in organic composition, our experimental eukaryotes increased the “richness” of their biomass when grown in the presence of grazers: all other things being equal, they became more palatable (also see 19 for an extended view of energy transfer through food chains [20], for a broader discussion on the interaction between external factor and compositional homeostasis, and [21] for the impact of growth rates on homeostasis of organic composition). Although the influence of phytoplankton prey composition on pelagic predators has been the subject of limited research (see 22 for a recent review), the opposite has seldom been considered. Our results clearly point towards a two-way interaction between predator and prey, seen as well in interactions among algal competitors [23], with implications for determining the energy available to trophic webs. 

Relative to cultures grown in the absence of grazers, our green algae and diatoms showed a strong increase in growth rate, perhaps because of rapid nutrient turnover associated with grazer metabolism. The increase was particularly pronounced for the diatoms grown in the presence of copepods. The dinoflagellate, instead, showed an avoidance strategy and encysted in the presence of either grazer. The cyanobacterial culture showed lower growth rate or, at 30 mM SO_4_^2-^, no difference. 

In previous research, Trommer et al. [24] grew a mixed assemblage of phytoplankton (dominated by diatoms and dinoflagellates) in the absence of grazers and in the presence of either *A. tonsa* or the rotifer *Brachionus plicatilis*, finding that phytoplankton biomass increased in the presence of micrometazoan grazers, (although results varied as a function of nutrient availability). Our results in experiments involving eukaryotic phytoplankton and copepod grazers are consistent with the report of Trommer et al. [24]. We find that protistan grazers also result in increased growth rates for eukaryotic phytoplankton, with the effect of ciliate grazing on our diatom population being significantly stronger than that of copepods. However, our experiments show that cyanobacteria actually show a decrease in growth rate when ciliate grazers are present. 

The potential evolutionary significance of these results emerges when we consider the Neoproterozoic radiation of protists capable of eating other protists (eukaryophagy). The ability to ingest bacteria-scale particles appears to be plesiomorphic among eukaryotes, but only a limited number of eukaryotic clades evolved eukaryophagy. Molecular clocks [25,26] suggest that the ciliates and other eukaryophagic clades radiated during the Neoproterozoic Era, long after the origin of the domain and, indeed, hundreds of millions of years after endosymbiosis led to the evolution of photosynthetic eukaryotes [26–28]. That eukaryotes rose to ecological prominence as primary producers in the oceans about when eukaryophagic protists radiated suggests a possible relationship between the two events [29,30]. We emphasize, once again, the limited range of our preliminary experiments, but stress that their implications warrant further research. Just as the advent of carnivory gave ecological impetus to Cambrian animal evolution (e.g., [31]), the Neoproterozoic radiation of eukaryophagic protists may have changed phytoplankton growth dynamics in a way that favored the expansion of eukaryotic phytoplankton in the oceans.

When small metazoan grazers expanded in the water columns of shelf seas is less clear. Fragmentary cuticular fossils indicate that total-group copepods had already differentiated by the Middle Cambrian Period [32]. Few fossils document the subsequent evolution of copepods [33], but there is a suggestion that the current ecological importance of calanoid copepods in pelagic food webs dates to the Mesozoic Era [34]. Interestingly, the effect of copepod grazers in our experiments was most pronounced in cultures of the diatom *T. weissflogii*. Copepod grazing was associated with decreased cell size in the diatom population, as well as a 4- to 6-fold increase in Si uptake. Pondevin et al. [35] found that Si uptake by *T. weissflogii* doubled when grown in media that previously contained diatoms and copepod grazers and interpreted the enhanced Si uptake as an inducible defence. Our results corroborate these results and extend them through the observation that grazing by a ciliate does not induce the same response.

Decreases in both mean diatom cell size and silica usage through the Cenozoic Era have been interpreted in terms of biophysical responses to directional changes in the marine environment, especially carbon dioxide and dissolved silica levels in surface oceans [36–39]. The experimental observation that both parameters change in response to copepod grazers adds nuance to the interpretation of observed evolutionary patterns. Based on biomechanical analyses, Hamm et al. [40] hypothesized that the silica frustules of diatoms resist crushing by mandibulate microarthropods such as copepods, and the correlation of silica uptake with the presence or absence of a copepod grazer is consistent with this hypothesis. 

Overall, our experiments document several distinct responses of phytoplankton to ciliate and copepod grazers. In some taxa, including the green algae and diatoms used here, grazing of either type induces a change in growth dynamics. Increased armour is another class of response, apparent in the increased silica uptake by diatoms exposed to copepod grazers and, as well, in the tendency of the dinoflagellate *Protoceratium reticulatum* to encyst. And toxin synthesis is a third response, effective against the copepod grazers in particular. Thus, even our limited range of experiments document the classes of response to grazing pressure well established among land plants. 

These experiments support the hypothesis that both protistan and metazoan grazing have influenced the evolution of marine phytoplankton through time. Continuing experiments on a wider phylogenetic sampling of phytoplankton, a greater range of chemical conditions, and an expanded roster of grazers should enable us to tease out the changing biological and physical factors that facilitated the observed long term evolutionary succession of phytoplankton in continental shelf and platform waters. Much remains to be done, but at this stage of understanding it seems that the debate about whether gazers, seawater chemistry or individual adaptations have shaped observed evolutionary patterns should be replaced by discussions of how all three have interacted though time to produce the evolutionary history inferred fossils, biomarker molecules, and molecular clocks.

## Supporting Information

Figure S1 **Growth in the presence of *A. tonsa*.** Specific growth rate of *T. suecica*, *T. weissflogii* and *Synechococcus* sp. cultured at 1 mM, 5 mM, 10 mM or 30 mM SO_4_^2-^ in the presence of the copepod *A. tonsa*. The error bars represent the standard deviation values calculated from at least three independent replicates.
(TIFF)

Figure S2 **Growth rate VS cellular volume Vs grazing *T. suecica*.** Specific growth rate expressed as a function of the cellular volume of *T. suecica*, cells cultured in the presence of *Euplotes* sp. or of *A. tonsa*, at 1 mM, 5 mM, 10 mM or 30 mM SO_4_^2-^. The error bars represent the standard deviation values calculate for at least three independent replicates.
(TIFF)

Figure S3 **Growth rate VS cellular volume Vs grazing *T. weissflogii*.** Specific growth rate expressed as a function of the cellular volume of *T. weissflogii*, cells cultured in the presence of *Euplotes* sp. or of *A. tonsa*, at 1 mM, 5 mM, 10 mM or 30 mM SO_4_^2-^. The error bars represent the standard deviation values calculate for at least three independent replicates.
(TIFF)

Figure S4 **Growth rate VS cellular volume Vs grazing *Synechococcus* sp.** Specific growth rate expressed as a function of the cellular volume of *Synechococcus* sp., cells cultured in the presence of *Euplotes* sp. or of *A. tonsa*, at 1 mM, 5 mM, 10 mM or 30 mM SO_4_^2-^. The error bars represent the standard deviation values calculate for at least three independent replicates.
(TIFF)

Figure S5 **Specific growth rate VS dry weight VS grazing *T. suecica*.** Specific growth rate expressed as a function of the cellular dry weight of *T. suecica* cells cultured in the presence of *Euplotes* sp. or of *A. tonsa*, at 1 mM, 5 mM, 10 mM or 30 mM SO_4_^2-^. The error bars represent the standard deviation values calculate for at least three independent replicates.
(TIFF)

Figure S6 **Specific growth rate VS dry weight VS grazing *T. weissflogii*.** Specific growth rate expressed as a function of the cellular dry weight of *T. weissflogii* cells cultured in the presence of *Euplotes* sp. or of *A. tonsa*, at 1 mM, 5 mM, 10 mM or 30 mM SO_4_^2-^. The error bars represent the standard deviation values calculate for at least three independent replicates.
(TIFF)

Figure S7 **Specific growth rate VS dry weight VS grazing *Synechococcus* sp.** Specific growth rate expressed as a function of the cellular dry weight of *T. weissflogii* cells cultured in the presence of *Euplotes* sp. or of *A. tonsa*, at 1 mM, 5 mM, 10 mM or 30 mM SO_4_^2-^. The error bars represent the standard deviation values calculate for at least three independent replicates.
(TIFF)

Figure S8 **Effect of grazers on the lipid:protein ratio.** Lipid to protein ratio of (A) *T. suecica*, (B) *T. weissflogii* and (C) *Synechococcus* sp. cells cultured at 1 mM, 5 mM, 10 mM or 30 mM SO_4_^2-^ in the presence of *Euplotes* sp. or *A. tonsa*. The value was normalized to the lipid to protein ratio calculated for the cells acclimated to 30 mM SO_4_^2-^ in the absence of grazers. The error bars represent the standard deviation values calculate for four independent replicates.
(TIFF)

Figure S9 **Effect of grazers on the carbohydrate:protein ratio.** Carbohydrate to protein ratio of (A) *T. suecica* and (B) *Synechococcus* sp. cells cultured in the presence of 1 mM, 5 mM, 10 mM or 30 mM SO_4_^2-^ and of *Euplotes* sp. or *A. tonsa*. The value was normalized to the carbohydrate to protein ratio calculated for the cells acclimated to 30 mM SO_4_^2-^ in the absence of grazers. The error bars represent the standard deviation values calculate for four independent replicates.
(TIFF)

Figure S10 **Effect of grazers on the carbohydrate:lipid ratio.** Carbohydrate to lipid ratio of (A) *T. suecica* and (B) *Synechococcus* sp. cells cultured in the presence of 1 mM, 5 mM, 10 mM or 30 mM SO_4_^2-^ and of *Euplotes* sp. or *A. tonsa*. The value was normalized to the carbohydrate to lipid ratio calculated for the cells acclimated to 30 mM SO_4_^2-^ in the absence of grazers. The error bars represent the standard deviation values calculate for four independent replicates.
(TIFF)

Figure S11 **Effect of grazers on the protein:silica ratio.** Protein to silicate ratio of *T. weissflogii* cells cultured in the presence of 1 mM, 5 mM, 10 mM or 30 mM SO_4_^2-^ and of *Euplotes* sp. or *A. tonsa*. The value was normalized to the protein to silicate ratio calculated for the cells acclimated to 30 mM SO_4_^2-^ in the absence of grazers. The error bars represent the standard deviation values calculate for four independent replicates.
(TIFF)

Table S1 **C:N:P:S. S=1.** Stechiometry of the main elements normalized to sulfur cell content of *T. suecica*, *T. weissflogii*, *Synechococcus* sp. cells cultured at 1 mM, 5 mM, 10 mM or 30 mM SO_4_^2-^ and in the presence of *Euplotes* sp. or *A. tonsa*. The results are shown as means ± standard deviations calculated for at least 4 independent replicates.
(DOCX)

Table S2 **Elemental stechiometry, elements normalized normalized to S cell content.** Stechiometry of the elements normalized to sulfur cell content of *T. suecica*, *T. weissflogii*, *Synechococcus* sp. cells cultured at 1 mM, 5 mM, 10 mM or 30 mM SO_4_^2-^ and in the presence of *Euplotes* sp. or *A. tonsa*. The results are shown as means ± standard deviations calculated for at least 4 independent replicates.
(DOCX)

Table S3 **Elements cell content.** Amount of elements in cells of *T. suecica*, *T. weissflogii*, *Synechococcus* sp. cells cultured at 1 mM, 5 mM, 10 mM or 30 mM SO_4_^2-^ and in the presence of *Euplotes* sp. or *A. tonsa*. The results are shown as means ± standard deviations calculated for at least 4 independent replicates.
(DOCX)

## References

[1] Falkowski PG, Katz ME, Knoll AH, Quigg A, Raven JA et al. (2004) The evolution of modern eukaryotic phytoplankton. Science 305: 354-360. doi:10.1126/science.1095964. PubMed: 15256663. 15256663

[2] Knoll AH, Summons RE, Waldbauer J, Zumberge J (2007) The geological succession of primary producers in the oceans. In Falkowski P Knoll AH The Evolution of Primary Producers in the Sea. Burlington: Elsevier pp. 133-163.

[3] Barton AD, Dutkiewicz S, Flierl G, Bragg J, Follows MJ (2010) Patterns of diversity in marine phytoplankton. Science 327: 1509-1511. doi:10.1126/science.1184961. PubMed: 20185684. 20185684

[4] Quigg A, Finkel ZV, Irwin AJ, Rosenthal Y, Ho TY et al. (2003) The evolutionary inheritance of elemental stoichiometry in marine phytoplankton. Nature 425: 291-294. doi:10.1038/nature01953. PubMed: 13679916. 13679916

[5] Ratti S, Knoll AH, Giordano M (2011) Did sulfate availability facilitate the evolutionary expansion of chlorophyll a+c phytoplankton in the oceans? Geobiology 29: 301-312. PubMed: 21627761. 10.1111/j.1472-4669.2011.00284.x 21627761

[6] Norici A, Hell R, Giordano M (2005) Sulfur and primary production in aquatic environments: an ecological perspective. Photosynth Res 86: 409-417. doi:10.1007/s11120-005-3250-0. PubMed: 16307310. 16307310

[7] Smetacek V (2012) Making sense of ocean biota: How evolution and biodiversity of land organisms differ from that of the plankton. J Biosci 37: 589–607. doi:10.1007/s12038-012-9240-4. PubMed: 22922185. 22922185

[8] Berges JA, Franklin DJ, Harrison PJ (2001) Evolution of an artificial seawater medium: improvements in enriched seawater, artificial water over the last two decades. J Phycol 37: 1138-1145. doi:10.1046/j.1529-8817.2001.01052.x.

[9] Leandro LF, Teegarden GJ, Roth PB, Wang Z, Doucette GJ (2010) The copepod *Calanus* *finmarchicus*: a potential vector for trophic transfer of the marine algal biotoxin, domoic acid. J Exp Mar Biol Ecol 382: 88-95. doi:10.1016/j.jembe.2009.11.002.

[10] Saage A, Vadstein O, Sommer U (2009) Feeding behaviour of adult *Centropages* *hamatus* (Copepoda, Calanoida): functional response and selective feeding experiments. J Sea Res 62: 16-21. doi:10.1016/j.seares.2009.01.002.

[11] Nejstgaard JC, Båmstedt U, Bagøien E, Solberg PT (1995) Algal constraints on copepod grazing. Growth state, toxicity, cell size, and season as regulating factors. ICES J Mar Sci 52: 347-357. doi:10.1016/1054-3139(95)80050-6.

[12] Palmucci M, Ratti S, Giordano M (2011) Carbon allocation as a function of nitrogen availability in marine phytoplankton: ecological and evolutionary implications. J Phycol 47: 313-323. doi:10.1111/j.1529-8817.2011.00963.x. 27021863

[13] Schreiber U, Schliwa U, Bilger W (1986) Continuous recording of photochemical and non-photochemical chlorophyll fluorescence quenching with a new type of modulation fluorometer. Photosynth Res 10: 51-62. doi:10.1007/BF00024185. 24435276

[14] Kramer DM, Johnson G, Kiirats O, Edwards KE (2004) New fluorescence parameters for the determination of QA redox state and excitation energy fluxes. Photosynth Res 79: 209-218. doi:10.1023/B:PRES.0000015391.99477.0d. PubMed: 16228395. 16228395

[15] Peterson GL (1977) Simplification of the protein assay method of Lowry which is more generally applicable. Anal Biochem 83: 346-356. doi:10.1016/0003-2697(77)90043-4. PubMed: 603028. 603028

[16] Domenighini A, Giordano M (2009) Fourier Transform Infrared spectroscopy of microalgae as a novel tool for biodiversity studies, species identification, and the assessment of water quality. J Phycol 45: 522–531. doi:10.1111/j.1529-8817.2009.00662.x. 27033830

[17] Giordano M, Kansiz MP, Heraud JP, Beardall JB, Wood B et al. (2001) Fourier transform infrared spectroscopy as a novel tool to investigate changes in intracellular macromolecular pools in the marine microalga *Chaetoceros* *muellerii* (Bacillariophyceae). J Phycol 37: 271-279. doi:10.1046/j.1529-8817.2001.037002271.x.

[18] Stehfest K, Toepel J, Wilhelm C (2005) The application of micro-FTIR spectroscopy to analyze nutrient stress-related changes in biomass composition of phytoplankton algae. Plant Physiol Biochem 43: 717-726. doi:10.1016/j.plaphy.2005.07.001. PubMed: 16122937. 16122937

[19] Hawlena D, Schmitz OJ (2010) Herbivore physiological response to predation risk and implications for ecosystem nutrient dynamics. Proc Natl Acad Sci U_S_A 107: 15503-15507. doi:10.1073/pnas.1009300107. PubMed: 20713698. 20713698 PMC2932623

[20] Giordano M (2013) Homeostasis: an underestimated focal point of ecology and evolution. Plant Sci 211: 92-101. doi:10.1016/j.plantsci.2013.07.008. PubMed: 23987815. 23987815

[21] Fanesi A, Raven JA, Giordano M (2013) Growth rate affects the responses of the green alga *Tetraselmis* *suecica* to external perturbations. Plant Cell Environ: ([MedlinePgn:]) doi:10.1111/pce.12176. PubMed: 23927015. 23927015

[22] Caron DA, Hutchins DA (2013) The effects of changing climate on microzooplankton grazing and community structure: drivers, predictions and knowledge gaps. J Plankton Res 35: 235-252. doi:10.1093/plankt/fbs091.

[23] Vardi A, Schatz D, Beeri K, Motro U, Sukenik A et al. (2002) Dinoflagellate-cyanobacterium communication may determine the composition of phytoplankton assemblage in a mesotrophic lake. Curr Biol 12: 1767-1772. doi:10.1016/S0960-9822(02)01217-4. PubMed: 12401172. 12401172

[24] Trommer G, Pondaven P, Siccha M, Stibor H (2012) Zooplankton-mediated nutrient limitation patterns in marine phytoplankton: an experimental approach with natural communities. Mar Ecol Prog Ser 449: 83-94. doi:10.3354/meps09508.

[25] Berney C, Pawlowski J (2006) A molecular time-scale for eukaryote evolution recalibrated with the continuous microfossil record. Proc R Soc Lond B 273: 1867-1872. doi:10.1098/rspb.2006.3537. PMC1634798 16822745

[26] Parfrey LW, Lahr DJ, Knoll AH, Katz LA (2011) Estimating the timing of early eukaryotic diversification with multigene molecular clocks. Proc Natl Acad Sci U_S_A 108: 13624-13629. doi:10.1073/pnas.1110633108. PubMed: 21810989. 21810989 PMC3158185

[27] Yoon HS, Hackett JD, Ciniglia C, Pinto G, Bhattacharya D (2004) A molecular timeline for the origin of photosynthetic eukaryotes. Mol Biol Evol 21: 809-818. doi:10.1093/molbev/msh075. PubMed: 14963099. 14963099

[28] Knoll AH, Javaux EJ, Hewitt D, Cohen P (2006) Eukaryotic organisms in Proterozoic oceans. Philos Trans R Soc Lond B 361: 1023-1038. doi:10.1098/rstb.2006.1843. PubMed: 16754612. 16754612 PMC1578724

[29] Knoll AH (2013) Paleobiological perspectives on early eukaryotic evolution. In Koonin E Keeling P Origin and evolution of eukaryotes. CSH Perspectives, Cold Spring Harbor Laboratory Press. In press 10.1101/cshperspect.a016121 PMC3941219 24384569

[30] Porter SM (2011) The rise of predators. Geology 39: 607-608. doi:10.1130/focus062011.1.

[31] Sperling EA, Frieder CA, Girguis PR, Levin LA, Knoll AH (2013) Oxygen, ecology, and the Cambrian radiation of animals. Proc Natl Acad Sci U_S_A 110: 13446-13451. doi:10.1073/pnas.1312778110. PubMed: 23898193. 23898193 PMC3746845

[32] Harvey THP, Vélez MI, Butterfield NJ (2012) Exceptionally preserved crustaceans from western Canada reveal a cryptic Cambrian radiation. Proc Natl Acad Sci U_S_A 109: 1589-1594. doi:10.1073/pnas.1203618109. PubMed: 22307616. 22307616 PMC3277126

[33] Selden PA, Huys R, Stephenson MH, Heward AP, Taylor PN (2010) Crustaceans from bitumen clast in Carboniferous glacial diamictite extend fossil record of copepods. Nature Comm. p. 1, Article number 50 Available: http://doi:10.1038/ncomms1049. 10.1038/ncomms1049 20975721

[34] Bradford-Grieve JM (2002) Colonization of the pelagic realm by calanoid copepods. Hydrobiologia 485: 223-244. doi:10.1023/A:1021373412738.

[35] Pondevan P, Gallinari M, Collet S, Bucciarelli E, Sarthou G et al. (2011) Grazing-induced changes in cell wall silicification in a marine diatom. Protist 158: 21-28. 10.1016/j.protis.2006.09.002 17081802

[36] Finkel ZV, Sebbo J, Feist-Burkhardt S, Irwin AJ, Katz ME et al. (2007) A universal driver of macroevolutionary change in the size of marine phytoplankton over the Cenozoic. Proc Natl Acad Sci U_S_A 104: 20416-20420. doi:10.1073/pnas.0709381104. PubMed: 18077334. 18077334 PMC2154445

[37] Finkel ZV, Matheson KA, Regan KS, Irwin AJ (2010) Genotypic and phenotypic variation in diatom silicification under paleo-oceanographic conditions. Geobiology 8: 433-445. doi:10.1111/j.1472-4669.2010.00250.x. PubMed: 20597991. 20597991

[38] Finkel ZV, Kotrc B (2010) Silica use trough time: macroevolutionary change in the morphology of the diatom frustule. Geomicrobiol J 27: 596-608. doi:10.1080/01490451003702941.

[39] Raven JA, Giordano M (2009) Biomineralization by photosynthetic organisms: evidence of coevolution of the organisms and their environment? Geobiology 7: 140-154. doi:10.1111/j.1472-4669.2008.00181.x. PubMed: 19207569. 19207569

[40] Hamm CE, Merkel R, Springer O, Jurkojc P, Maier C et al. (2003) Architecture and material properties of diatom shells provide effective mechanical protection. Nature 421: 841-843. doi:10.1038/nature01416. PubMed: 12594512. 12594512

