# Supplementary figures and images for: Grazers and Phytoplankton Growth in the Oceans: an Experimental and Evolutionary Perspective
Source: PLoS One. 2013 Oct 24;8(10):e77349. doi: 10.1371/journal.pone.0077349 (PMC3811990; doi:10.1371/journal.pone.0077349)

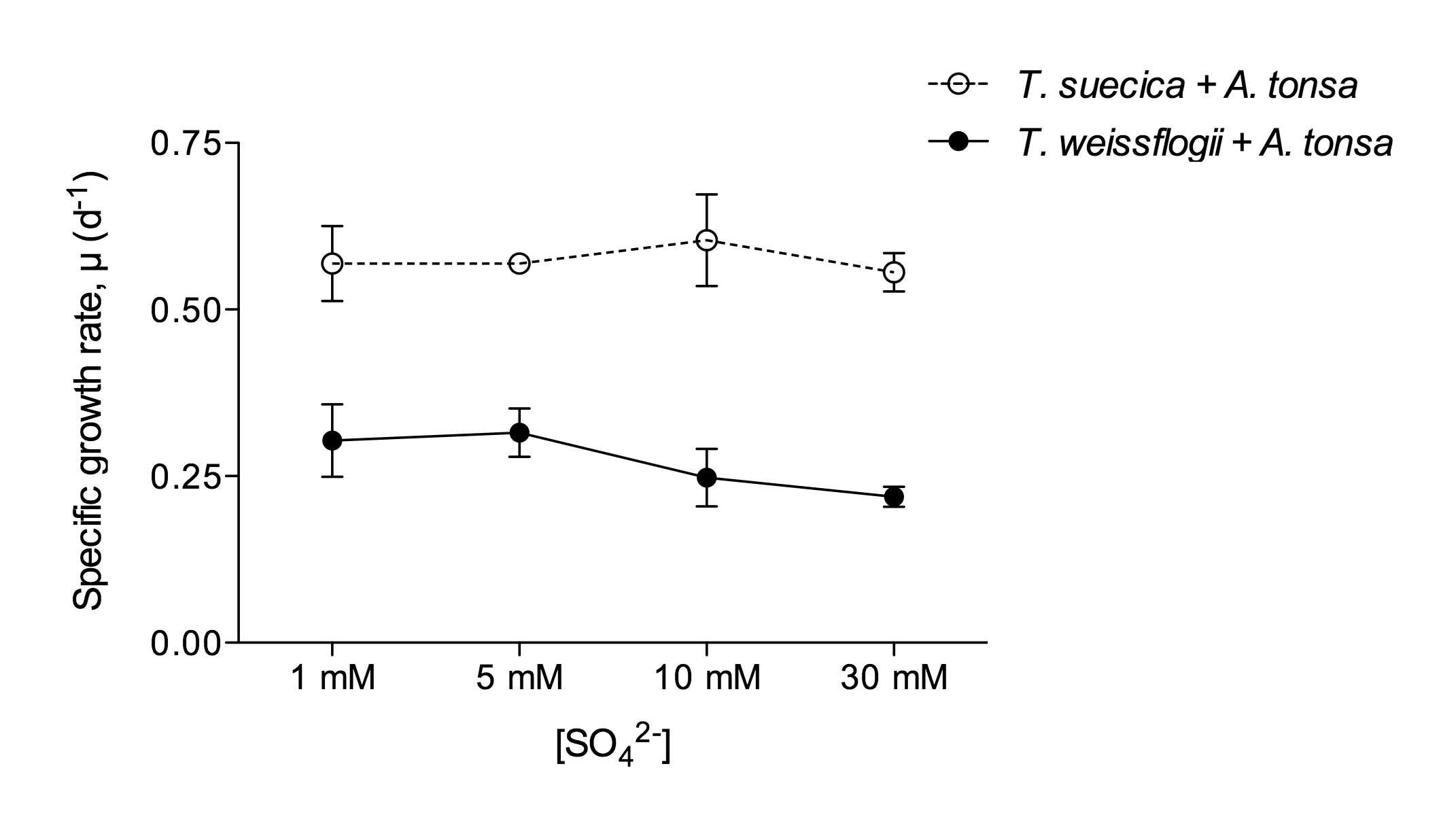

Supplement: Figure S1 — Growth in the presence of A. tonsa. Specific growth rate of T. suecica, T. weissflogii and Synechococcus sp. cultured at 1 mM, 5 mM, 10 mM or 30 mM SO4 2- in the presence of the copepod A. tonsa. The error bars represent the standard deviation values calculated from at least three independent replicates. (TIFF) [file pone.0077349.s001.tiff]

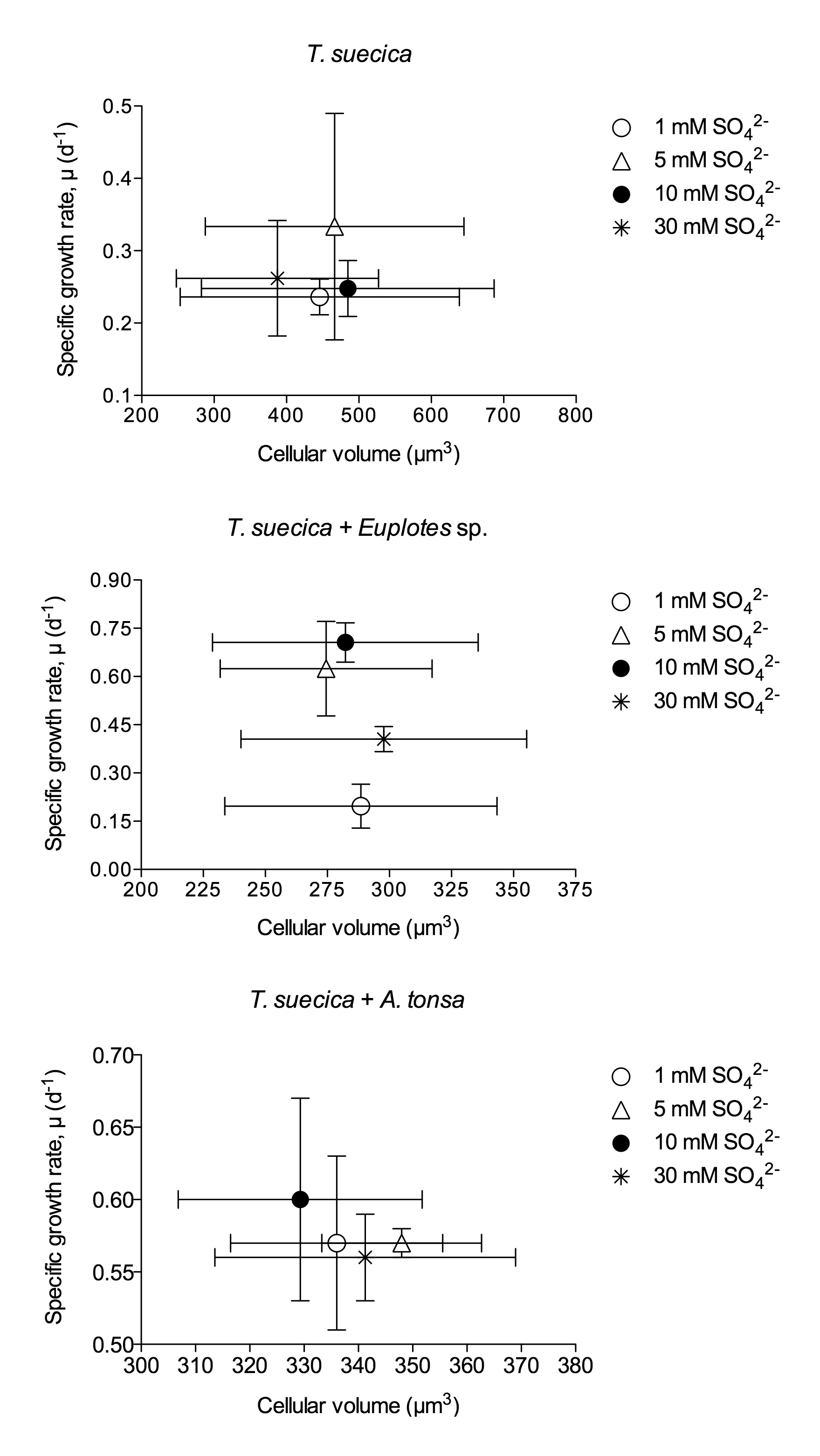

Supplement: Figure S2 — Growth rate VS cellular volume Vs grazing T. suecica. Specific growth rate expressed as a function of the cellular volume of T. suecica, cells cultured in the presence of Euplotes sp. or of A. tonsa, at 1 mM, 5 mM, 10 mM or 30 mM SO4 2-. The error bars represent the standard deviation values calculate for at least three independent replicates. (TIFF) [file pone.0077349.s002.tiff]

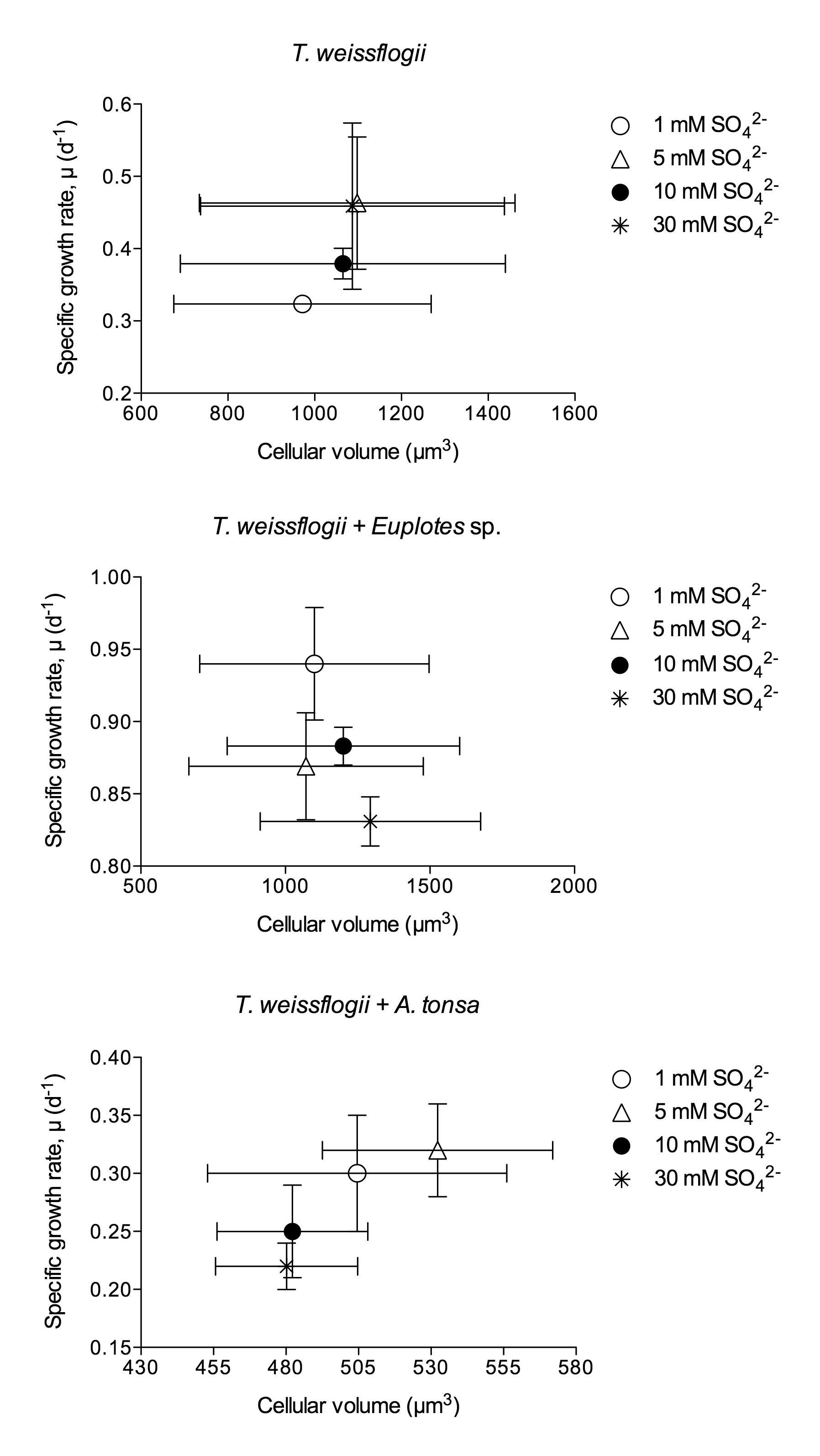

Supplement: Figure S3 — Growth rate VS cellular volume Vs grazing T. weissflogii. Specific growth rate expressed as a function of the cellular volume of T. weissflogii, cells cultured in the presence of Euplotes sp. or of A. tonsa, at 1 mM, 5 mM, 10 mM or 30 mM SO4 2-. The error bars represent the standard deviation values calculate for at least three independent replicates. (TIFF) [file pone.0077349.s003.tiff]

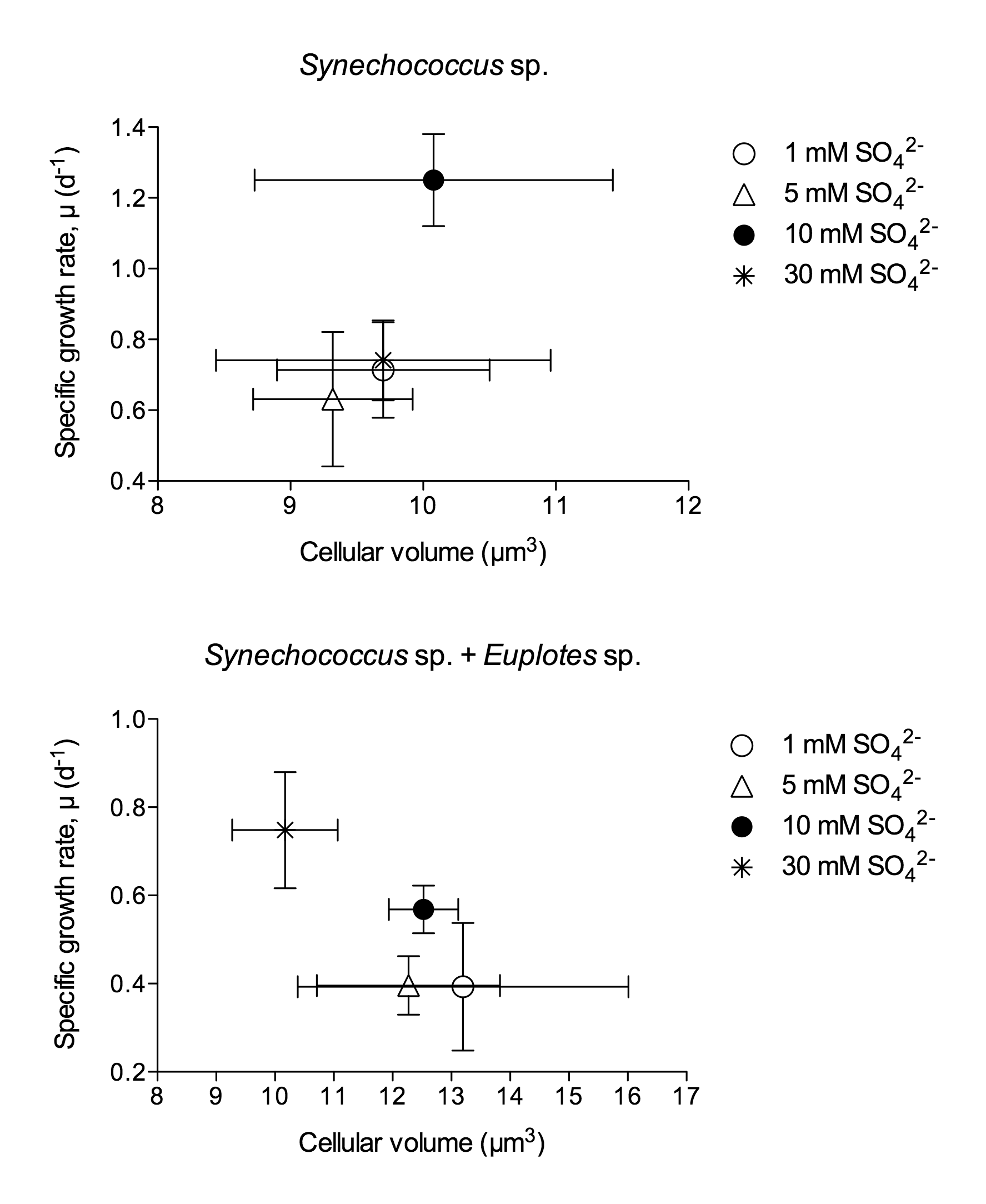

Supplement: Figure S4 — Growth rate VS cellular volume Vs grazing Synechococcus sp. Specific growth rate expressed as a function of the cellular volume of Synechococcus sp., cells cultured in the presence of Euplotes sp. or of A. tonsa, at 1 mM, 5 mM, 10 mM or 30 mM SO4 2-. The error bars represent the standard deviation values calculate for at least three independent replicates. (TIFF) [file pone.0077349.s004.tiff]

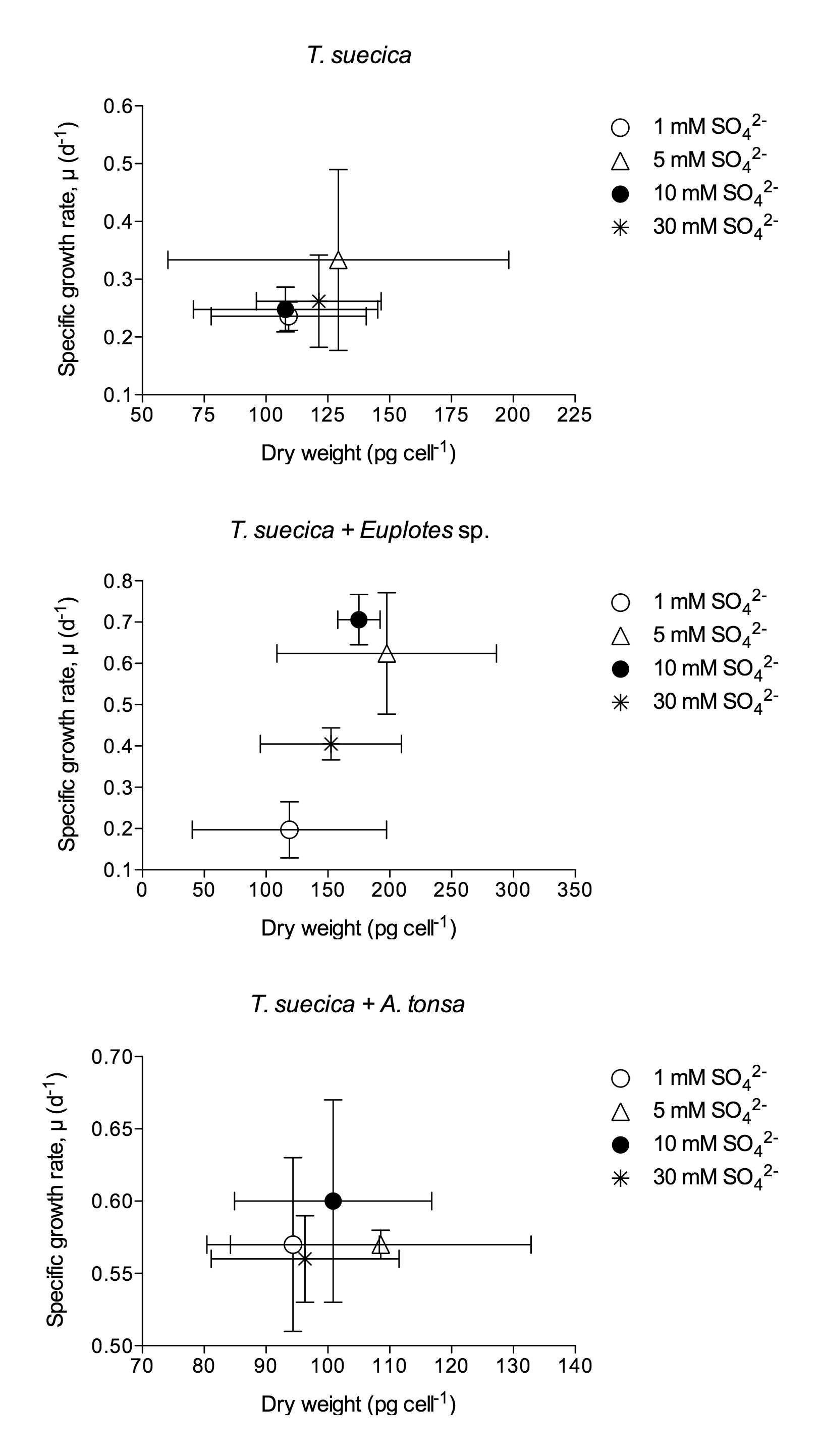

Supplement: Figure S5 — Specific growth rate VS dry weight VS grazing T. suecica. Specific growth rate expressed as a function of the cellular dry weight of T. suecica cells cultured in the presence of Euplotes sp. or of A. tonsa, at 1 mM, 5 mM, 10 mM or 30 mM SO4 2-. The error bars represent the standard deviation values calculate for at least three independent replicates. (TIFF) [file pone.0077349.s005.tiff]

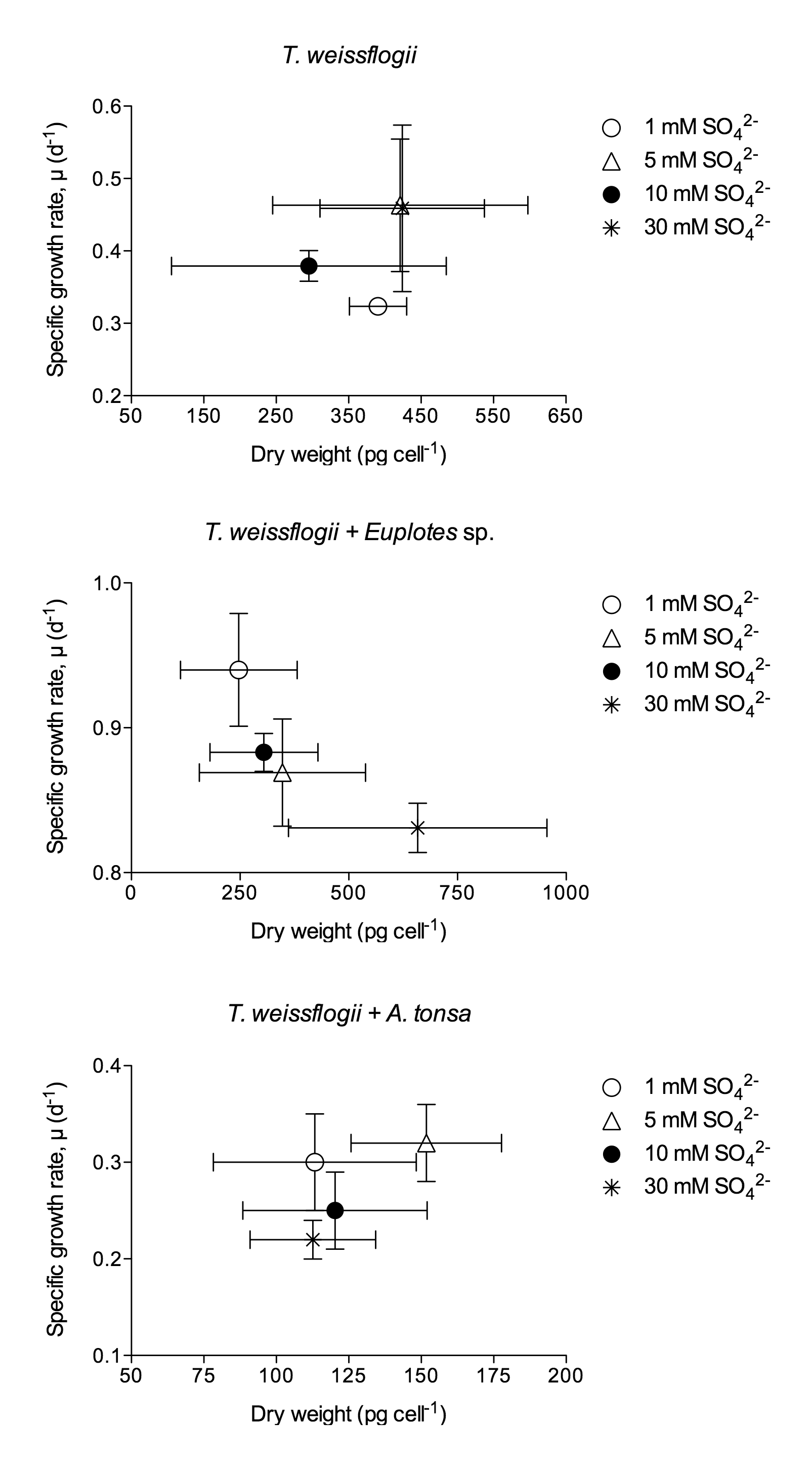

Supplement: Figure S6 — Specific growth rate VS dry weight VS grazing T. weissflogii. Specific growth rate expressed as a function of the cellular dry weight of T. weissflogii cells cultured in the presence of Euplotes sp. or of A. tonsa, at 1 mM, 5 mM, 10 mM or 30 mM SO4 2-. The error bars represent the standard deviation values calculate for at least three independent replicates. (TIFF) [file pone.0077349.s006.tiff]

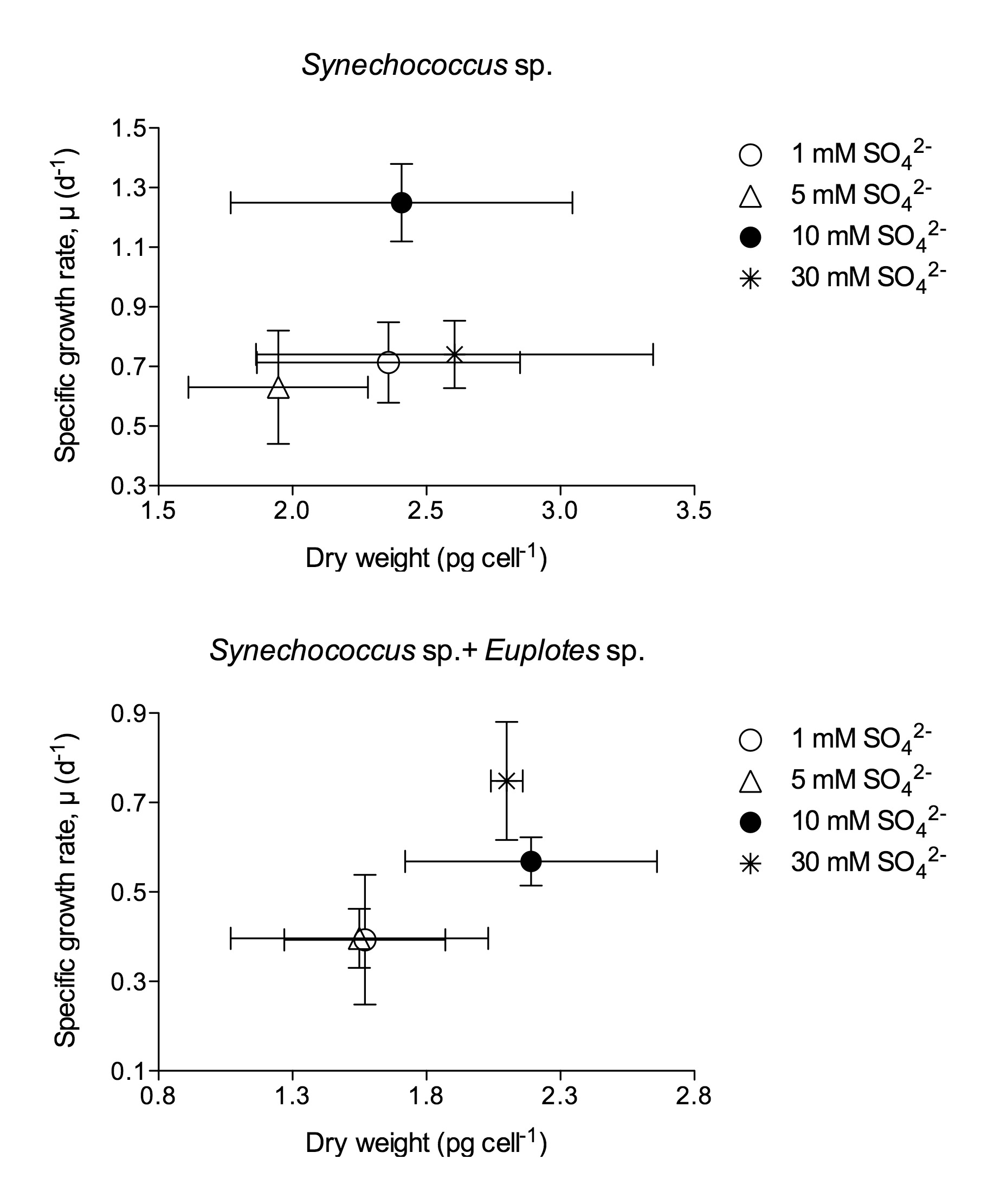

Supplement: Figure S7 — Specific growth rate VS dry weight VS grazing Synechococcus sp. Specific growth rate expressed as a function of the cellular dry weight of T. weissflogii cells cultured in the presence of Euplotes sp. or of A. tonsa, at 1 mM, 5 mM, 10 mM or 30 mM SO4 2-. The error bars represent the standard deviation values calculate for at least three independent replicates. (TIFF) [file pone.0077349.s007.tiff]

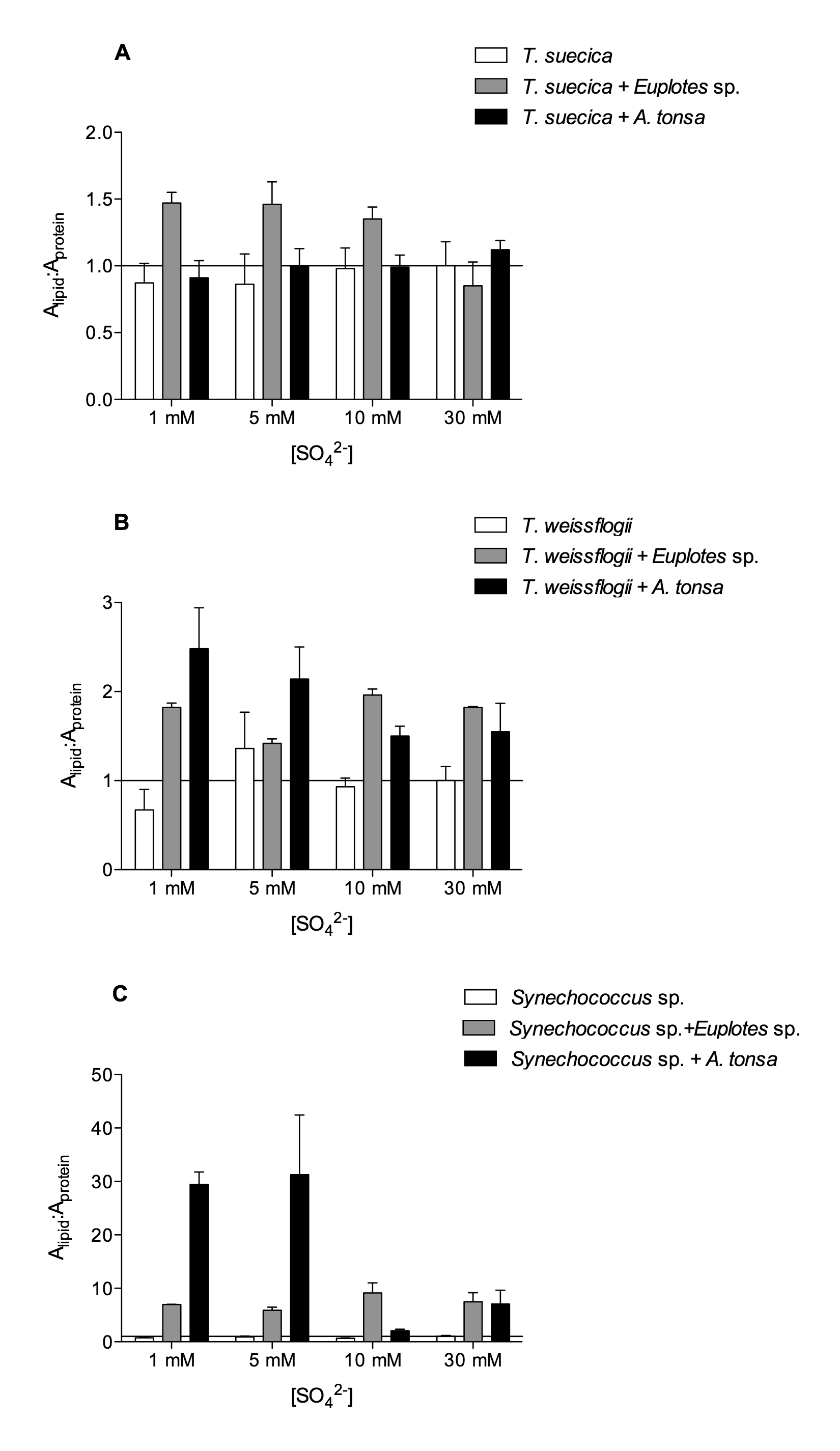

Supplement: Figure S8 — Effect of grazers on the lipid:protein ratio. Lipid to protein ratio of (A) T. suecica, (B) T. weissflogii and (C) Synechococcus sp. cells cultured at 1 mM, 5 mM, 10 mM or 30 mM SO4 2- in the presence of Euplotes sp. or A. tonsa. The value was normalized to the lipid to protein ratio calculated for the cells acclimated to 30 mM SO4 2- in the absence of grazers. The error bars represent the standard deviation values calculate for four independent replicates. (TIFF) [file pone.0077349.s008.tiff]

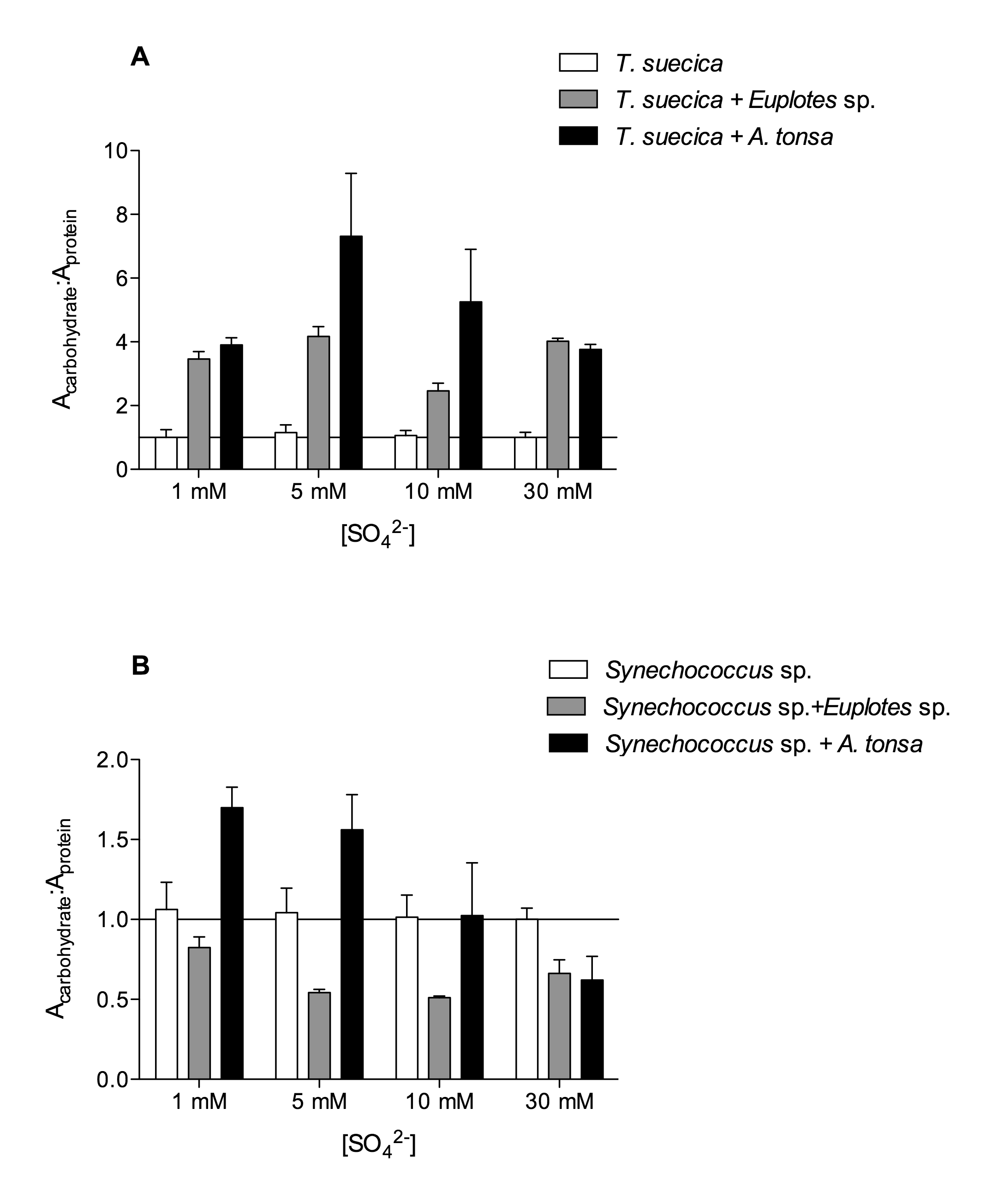

Supplement: Figure S9 — Effect of grazers on the carbohydrate:protein ratio. Carbohydrate to protein ratio of (A) T. suecica and (B) Synechococcus sp. cells cultured in the presence of 1 mM, 5 mM, 10 mM or 30 mM SO4 2- and of Euplotes sp. or A. tonsa. The value was normalized to the carbohydrate to protein ratio calculated for the cells acclimated to 30 mM SO4 2- in the absence of grazers. The error bars represent the standard deviation values calculate for four independent replicates. (TIFF) [file pone.0077349.s009.tiff]

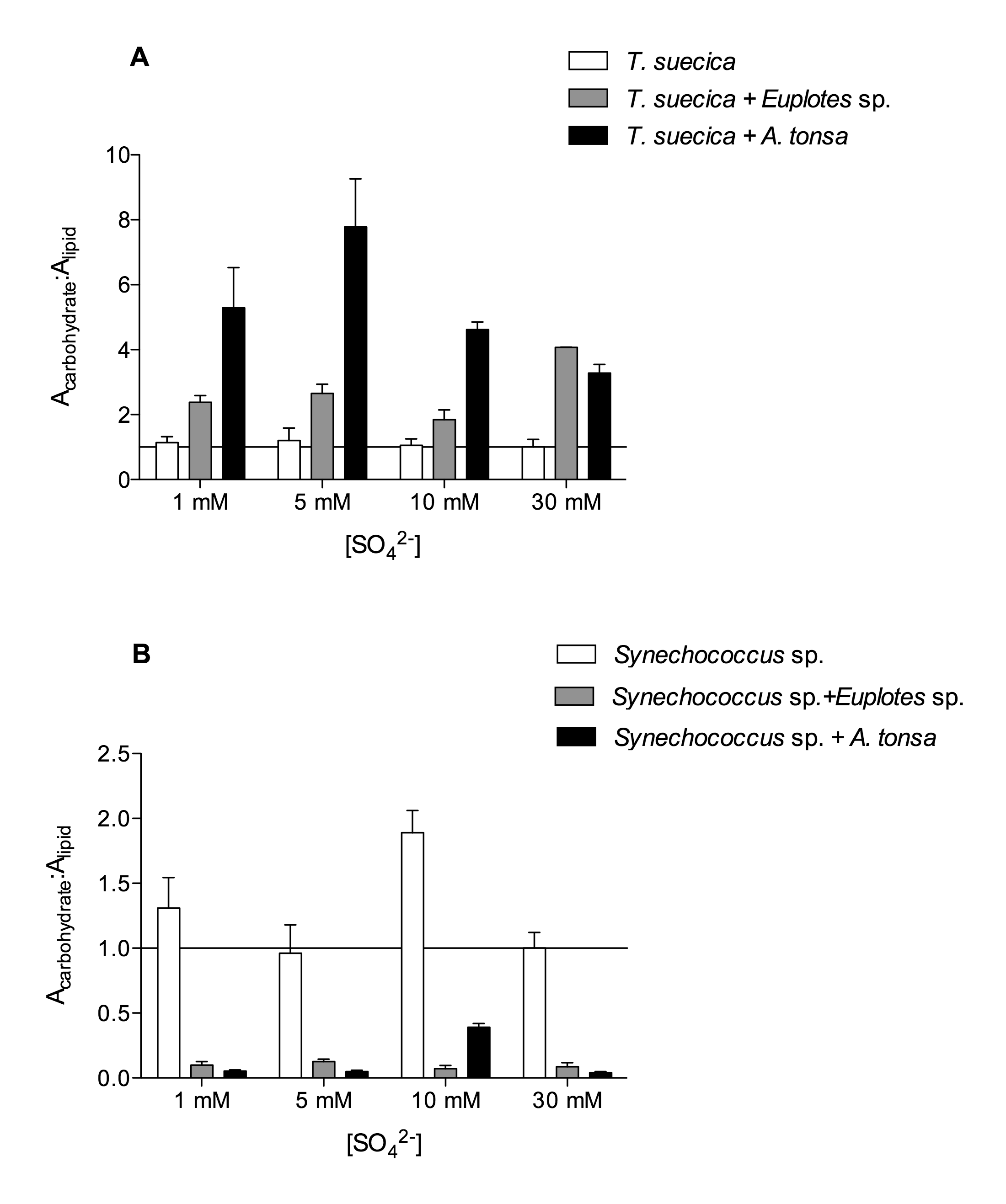

Supplement: Figure S10 — Effect of grazers on the carbohydrate:lipid ratio. Carbohydrate to lipid ratio of (A) T. suecica and (B) Synechococcus sp. cells cultured in the presence of 1 mM, 5 mM, 10 mM or 30 mM SO4 2- and of Euplotes sp. or A. tonsa. The value was normalized to the carbohydrate to lipid ratio calculated for the cells acclimated to 30 mM SO4 2- in the absence of grazers. The error bars represent the standard deviation values calculate for four independent replicates. (TIFF) [file pone.0077349.s010.tiff]

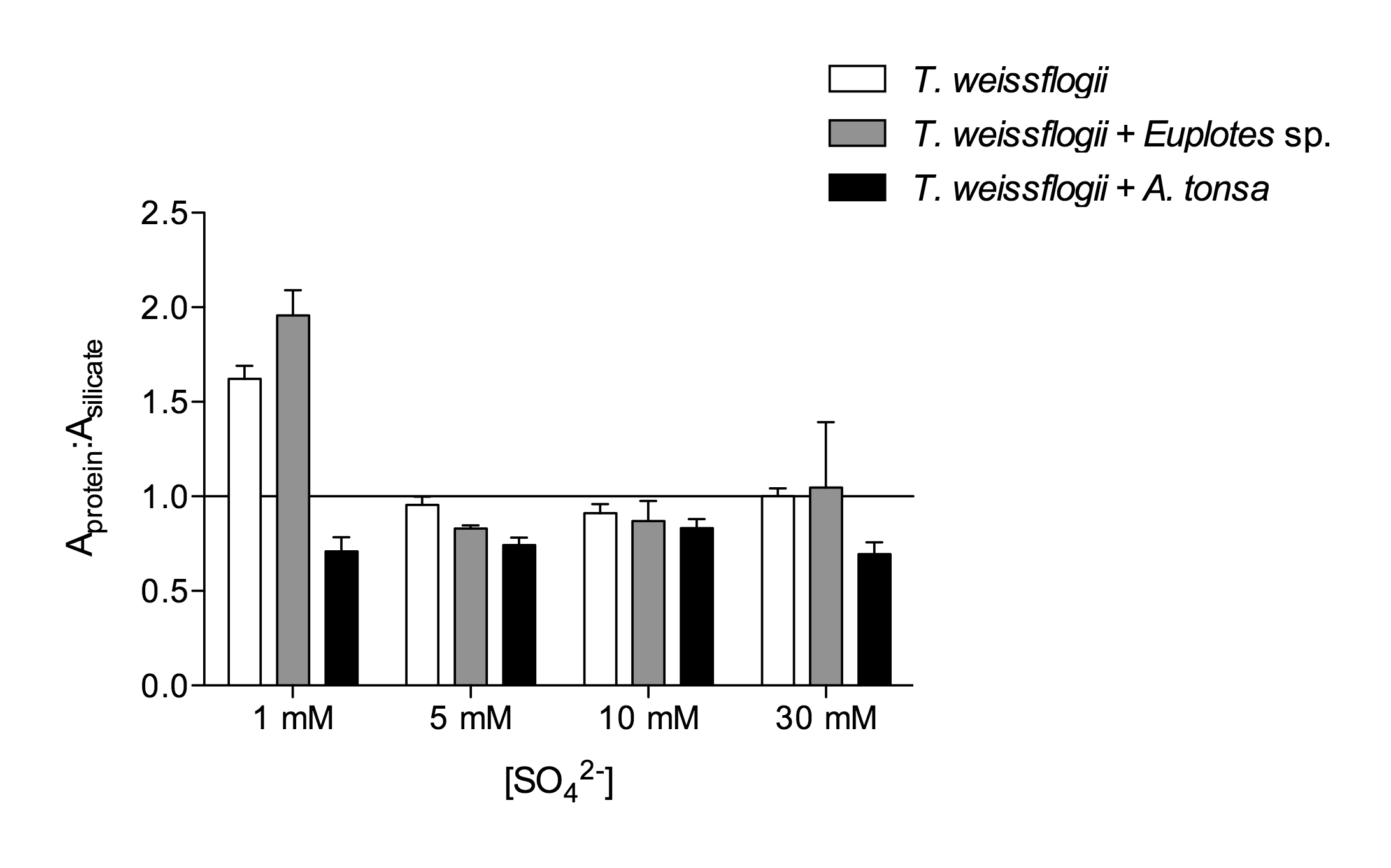

Supplement: Figure S11 — Effect of grazers on the protein:silica ratio. Protein to silicate ratio of T. weissflogii cells cultured in the presence of 1 mM, 5 mM, 10 mM or 30 mM SO4 2- and of Euplotes sp. or A. tonsa. The value was normalized to the protein to silicate ratio calculated for the cells acclimated to 30 mM SO4 2- in the absence of grazers. The error bars represent the standard deviation values calculate for four independent replicates. (TIFF) [file pone.0077349.s011.tiff]
