# Supplementary material for: Grazers and Phytoplankton Growth in the Oceans: an Experimental and Evolutionary Perspective
Source: PLoS One. 2013 Oct 24;8(10):e77349. doi: 10.1371/journal.pone.0077349 (PMC3811990; doi:10.1371/journal.pone.0077349)
Supplement: Table S1 — C:N:P:S. S=1. Stechiometry of the main elements normalized to sulfur cell content of T. suecica, T. weissflogii, Synechococcus sp. cells cultured at 1 mM, 5 mM, 10 mM or 30 mM SO4 2- and in the presence of Euplotes sp. or A. tonsa. The results are shown as means ± standard deviations calculated for at least 4 independent replicates. (DOCX) [file pone.0077349.s012.docx]

Table S1: C:N:P:S. S=1

|  | [SO_4_^2-^] |  | Element mass ratio | | | |
| --- | --- | --- | --- | --- | --- | --- |
|  | *mM* |  | C | N | P | S |
| *T. suecica* | 5 |  | 62.7 ± 3.69 | 11.3 ± 1.77 | 0.50 ± 0.27 | 1.00 ± 0.55 |
|  | 30 |  | 71.5 ± 4.18 | 12.9 ± 1.27 | 0.68 ± 0.14 | 1.00 ± 0.21 |
|  |  |  |  |  |  |  |
| *T. suecica* + *Euplotes* sp. | 5 |  | 52.4 ± 8.69 | 8.82 ± 0.53 | 0.25 ± 0.14 | 1.00 ± 0.29 |
|  | 30 |  | 45.2 ± 3.12 | 6.63 ± 1.27 | 0.51 ± 0.28 | 1.00 ± 0.49 |
|  |  |  |  |  |  |  |
| *T. suecica* +  *A. tonsa* | 5 |  | 79.9 ± 5.37 | 14.8 ± 1.12 | 0.72 ± 0.09 | 1.00 ± 0.15 |
|  | 30 |  | 82.3 ± 3.55 | 15.6 ± 1.02 | 0.74 ± 0.10 | 1.00 ± 0.30 |
|  |  |  |  |  |  |  |
| *T. weissflogii* | 5 |  | 63.8 ± 4.79 | 8.90 ± 0.93 | 0.78 ± 0.33 | 1.00 ± 0.42 |
|  | 30 |  | 174 ± 2.05 | 7.84 ± 0.68 | 0.38 ± 0.10 | 1.00 ± 0.27 |
|  |  |  |  |  |  |  |
| *T. weissflogii* + *Euplotes* sp. | 5 |  | 50.7 ± 6.74 | 8.28 ± 0.43 | 0.52 ± 0.19 | 1.00 ± 0.00 |
|  | 30 |  | 55.3 ± 2.95 | 8.46 ± 0.35 | 0.54 ± 0.00 | 1.00 ± 0.00 |
|  |  |  |  |  |  |  |
| *T. weissflogii* +  *A. tonsa* | 5 |  | 39.5 ± 2.91 | 5.73 ± 0.64 | 0.82 ± 0.29 | 1.00 ± 0.25 |
|  | 30 |  | 33.5 ± 3.73 | 5.39 ± 0.25 | 1.21 ± 0.54 | 1.00 ± 0.09 |
|  |  |  |  |  |  |  |
| *Synechococcus* sp. | 5 |  | 91.3 ± 4.23 | 17.1 ± 2.05 | 4.43 ± 0.76 | 1.00 ± 0.19 |
|  | 30 |  | 73.4 ± 2.75 | 14.1 ± 1.18 | 3.66 ± 1.04 | 1.00 ± 0.28 |
|  |  |  |  |  |  |  |
| *Synechococcus* sp. +  *Euplotes* sp. | 5 |  | 906 ± 36.4 | 200 ± 9.49 | 3.66 ± 2.13 | 1.00 ± 0.58 |
|  | 30 |  | 86.9 ± 7.90 | 17.5 ± 1.27 | 1.63 ± 1.09 | 1.00 ± 0.12 |
|  |  |  |  |  |  |  |
| *Synechococcus* sp. +  *A. tonsa* | 5 |  | 144 ± 12.6 | 21.4 ± 2.02 | 2.12 ± 0.73 | 1.00 ± 0.29 |
|  | 30 |  | 11.2 ± 1.06 | 8.30 ± 2.13 | 3.29 ± 1.97 | 1.00 ± 0.28 |
