# Supplementary material for: Grazers and Phytoplankton Growth in the Oceans: an Experimental and Evolutionary Perspective
Source: PLoS One. 2013 Oct 24;8(10):e77349. doi: 10.1371/journal.pone.0077349 (PMC3811990; doi:10.1371/journal.pone.0077349)
Supplement: Table S2 — Elemental stechiometry, elements normalized normalized to S cell content. Stechiometry of the elements normalized to sulfur cell content of T. suecica, T. weissflogii, Synechococcus sp. cells cultured at 1 mM, 5 mM, 10 mM or 30 mM SO4 2- and in the presence of Euplotes sp. or A. tonsa. The results are shown as means ± standard deviations calculated for at least 4 independent replicates. (DOCX) [file pone.0077349.s013.docx]

Table S2: Elemental stechiometry, elements normalized normalized to S cell content

| Species | [SO_4_^2-^] |  | Element mass ratio | | | | | | | | | | | | | | | |
| --- | --- | --- | --- | --- | --- | --- | --- | --- | --- | --- | --- | --- | --- | --- | --- | --- | --- | --- |
|  | *mM* |  |  |  |  |  |  |  |  |  |  |  |  |  |  |  |  |  |
|  |  |  | Si | C | N | P | S | K | Ca | Cr | Mn | Fe | Ni | Cu | Zn | Br | Sr | Pb |
|  |  |  |  |  |  |  |  |  |  |  |  |  |  |  |  |  |  |  |
| *T. suecica* | 5 |  | n.d. | 62.77 (3.694) | 11.36 (1.77) | 0.500 (0.274) | 1.000 (0.549) | 0.405 (0.217) | 1.091 (0.585) | 0.000 (0.000) | 0.011 (0.006) | 0.176 (0.094) | 0.000 (0.000) | 0.001 (0.001) | 0.013 (0.007) | 0.002 (0.001) | 1.733 (0.925) | 0.001 (0.000) |
|  | 30 |  | n.d. | 71.50 (4.180) | 12.95 (1.278) | 0.684 (0.143) | 1.000 (0.209) | 0.408 (0.087) | 0.639 (0.133) | 0.002 (0.000) | 0.007 (0.002) | 0.400 (0.084) | 0.000 (0.000) | 0.001 (0.000) | 0.009 (0.002) | 0.003 (0.001) | 0.676 (0.141) | 0.000 (0.000) |
| *T. suecica*  +  *Euplotes* sp. | 5 |  | n.d. | 52.42 (8.687) | 8.816 (0.531) | 0.250 (0.145) | 1.000 (0.294) | 0.134 (0.126) | 0.997 (0.639) | 0.0.00 (0.000) | 0.005 (0.006) | 0.086 (0.066) | 0.000 (0.000) | 0.001 (0.000) | 0.009 (0.004) | 0.002 (0.001) | 1.104 (1.466) | 0.000 (0.000) |
|  | 30 |  | n.d. | 45.17 (3.122) | 6.634 (1.275) | 0.512 (0.284) | 1.000 (0.492) | 0.128 (0.104) | 1.409 (0.835) | 0.000 (0.000) | 0.006 (0.004) | 0.077 (0.034) | 0.000 (0.000) | 0.001 (0.001) | 0.012 (0.005) | 0.001 (0.001) | 1.541 (0.956) | 0.000 (0.000) |
| *T. suecica*  *+*  *A. tonsa* | 5 |  | n.d. | 79.95 (5.370) | 14.84 (1.124) | 0.722 (0.089) | 1.000 (0.155) | 0.024 (0.016) | 0.349 (0.045) | 0.000 (0.000) | 0.004 (0.002) | 0.366 (0.108) | 0.000 (0.000) | 0.002 (0.001) | 0.014 (0.002) | 0.017 (0.016) | 0.017 (0.011) | 0.001 (0.001) |
|  | 30 |  | n.d. | 82.26 (3.551) | 15.46 (1.022) | 0.743 (0.103) | 1.000 (0.305) | 0.008 (0.003) | 0.355 (0.097) | 0.003 (0.001) | 0.006 (0.004) | 0.595 (0.060) | 0.000 (0.000) | 0.002 (0.001) | 0.015 (0.005) | 0.002 (0.002) | 0.168 (0.189) | 0.002 (0.001) |
|  |  |  |  |  |  |  |  |  |  |  |  |  |  |  |  |  |  |  |
| *T. weissflogii* | 5 |  | 11.38 (4.777) | 63.82 (4.79) | 8.995 (0.933) | 0.781 (0.327) | 1.000 (0.418) | 0.283 (0.118) | 0.162 (0.070) | 0.000 (0.000) | 0.012 (0.005) | 0.332 (0.139) | 0.000 (0.00) | 0.001 (0.001) | 0.009 (0.004) | 0.016 (0.007) | 0.006 (0.002) | 0.001 (0.001) |
|  | 30 |  | 8.679 (2.341) | 174.5 (2.05) | 7.838 (0.685) | 0.380 (0.104) | 1.000 (0.269) | 0.214 (0.058) | 0.129 (0.036) | 0.002 (0.000) | 0.013 (0.003) | 0.447 (0.122) | 0.001 (0.001) | 0.001 (0.000) | 0.009 (0.003) | 0.014 (0.004) | 0.006 (0.002) | 0.002 (0.000) |
| *T. weissflogii*  +  *Euplotes* sp. | 5 |  | 7.988 (0.000) | 50.67 (6.743) | 8.281 (0.436) | 0.518 (0.000) | 1.000 (0.00) | 0.152 (0.000) | 0.316 (0.000) | 0.000 (0.000) | 0.012 (0.000) | 0.289 (0.000) | 0.000 (0.000) | 0.001 (0.000) | 0.015 (0.000) | 0.010 (0.000) | 0.162 (0.000) | 0.002 (0.000) |
|  | 30 |  | 9.439 (0.000) | 55.23 (2.950) | 8.458 (0.348) | 0.537 (0.000) | 1.000 (0.000) | 0.209 (0.000) | 0.257 (0.000) | 0.001 (0.000) | 0.013 (0.000) | 0.323 (0.000) | 0.000 (0.000) | 0.001 (0.000) | 0.016 (0.000) | 0.008 (0.000) | 0.189 (0.000) | 0.002 (0.000) |
| *T. weissflogii*  +  *A. tonsa* | 5 |  | 7.098 (1.009) | 39.46 (2.910) | 5.729 (0.636) | 0.821 (0.295) | 1.000 (0.254) | 0.077 (0.055) | 0.094 (0.019) | 0.000 (0.000) | 0.006 (0.001) | 0.287 (0.033) | 0.000 (0.000) | 0.001 (0.000) | 0.013 (0.002) | 0.058 (0.110) | 0.006 (0.010) | 0.001 (0.000) |
|  | 30 |  | 9.005 (1.075) | 33.48 (3.373) | 5.390 (0.249) | 1.208 (0.541) | 1.000 (0.088) | 0.104 (0.060) | 0.211 (0.121) | 0.001 (0.000) | 0.009 (0.002) | 0.434 (0.103) | 0.000 (0.000) | 0.001 (0.000) | 0.014 (0.003) | 0.016 (0.023) | 0.002 (0.001) | 0.001 (0.000) |
|  |  |  |  |  |  |  |  |  |  |  |  |  |  |  |  |  |  |  |
| *Synechococcus* sp. | 5 |  | n.d. | 91.33 (4.227) | 17.066 (2.050) | 4.426 (0.762) | 1.000 (0.189) | 0.290 (0.050) | 0.442 (0.076) | 0.002 (0.000) | 0.010 (0.002) | 1.050 (0.182) | 0.001 (0.000) | 0.002 (0.000) | 0.012 (0.003) | 0.001 (0.000) | 0.028 (0.005) | 0.008 (0.002) |
|  | 30 |  | n.d. | 73.43 (2.754) | 14.15 (1.182) | 3.661 (1.044) | 1.000 (0.285) | 0.211 (0.060) | 0.240 (0.068) | 0.009 (0.003) | 0.009 (0.003) | 1.043 (0.297) | 0.002 (0.001) | 0.002 (0.001) | 0.015 (0.004) | 0.001 (0.000) | 0.018 (0.005) | 0.008 (0.002) |
| *Synechococcus* sp.  +  *Euplotes* sp. | 5 |  | n.d. | 906.7 (36.39) | 200.1 (9.492) | 3.662 (2.133) | 1.000 (0.583) | 3.005 (3.328) | 17.53 (23.60) | 0.000 (0.000) | 0.028 (0.020) | 3.462 (1.363) | 0.000 (0.000) | 0.058 (0.073) | 0.168 (0.163) | 0.106 (0.054) | 0.417 (0.283) | 0.012 (0.008) |
|  | 30 |  | n.d. | 86.92 (7.899) | 17.57 (1.271) | 1.629 (1.088) | 1.000 (0.120) | 0.185 (0.082) | 0.498 (0.486) | 0.000 (0.000) | 0.001 (0.002) | 0.125 (0.193) | 0.000 (0.000) | 0.002 (0.001) | 0.007 (0.001) | 0.017 (0.016) | 0.017 (0.011) | 0.002 (0.002) |
| *Synechococcus* sp.  +  *A. tonsa* | 5 |  | n.d. | 144.7 (12.63) | 21.43 (2.021) | 2.124 (0.730) | 1.000 (0.287) | 0.033 (0.001) | 0.220 (0.130) | 0.002 (0.001) | 0.014 (0.004) | 0.952 (0.075) | 0.001 (0.000) | 0.002 (0.001) | 0.018 (0.000) | 0.002 (0.001) | 0.003 (0.001) | 0.000 (0.000) |
|  | 30 |  | n.d. | 11.216 (1.057) | 8.301 (2.135) | 3.287 (1.974) | 1.000 (0.283) | 0.064 (0.041) | 0.687 (0.395) | 0.010 (0.007) | 0.017 (0.009) | 4.690 (2.575) | 0.001 (0.001) | 0.005 (0.005) | 0.036 (0.028) | 0.003 (0.003) | 0.010 (0.003) | 0.002 (0.001) |
