# Supplementary material for: Grazers and Phytoplankton Growth in the Oceans: an Experimental and Evolutionary Perspective
Source: PLoS One. 2013 Oct 24;8(10):e77349. doi: 10.1371/journal.pone.0077349 (PMC3811990; doi:10.1371/journal.pone.0077349)
Supplement: Table S3 — Elements cell content. Amount of elements in cells of T. suecica, T. weissflogii, Synechococcus sp. cells cultured at 1 mM, 5 mM, 10 mM or 30 mM SO4 2- and in the presence of Euplotes sp. or A. tonsa. The results are shown as means ± standard deviations calculated for at least 4 independent replicates. (DOCX) [file pone.0077349.s014.docx]

Table S3. Elements cell content

| Species | [SO_4_^2-^] |  | Element cell content (*fg E • pg dry weight^-1^*) | | | | | | | | | | | | | | | |
| --- | --- | --- | --- | --- | --- | --- | --- | --- | --- | --- | --- | --- | --- | --- | --- | --- | --- | --- |
|  | *mM* |  |  |  |  |  |  |  |  |  |  |  |  |  |  |  |  |  |
|  |  |  | Si | C | N | P | S | K | Ca | Cr | Mn | Fe | Ni | Cu | Zn | Br | Sr | Pb |
|  |  |  |  |  |  |  |  |  |  |  |  |  |  |  |  |  |  |  |
| *T. suecica* | 5 |  | n.d. | 433 ±25.5 | 78.5 ±12.2 | 3.45 ± 1.89 | 6.91 ± 3.79 | 2.80 ± 1.50 | 7.54 ± 4.04 | 0.00 ± 0.00 | 0.07 ± 0.04 | 1.22 ± 0.65 | 0.00 ± 0.00 | 0.01 ± 0.00 | 0.09 ± 0.05 | 0.01 ± 0.01 | 11.98 ± 6.39 | 0.01 ± 0.00 |
|  | 30 |  | n.d. | 433 ± 25.4 | 78.5 ± 7.70 | 4.15 ± 0.87 | 6.07 ± 1.27 | 2.47 ± 0.53 | 3.88 ± 0.81 | 0.01 ± 0.00 | 0.04 ± 0.01 | 2.43 ± 0.51 | 0.00 ± 0.00 | 0.01 ± 0.00 | 0.06 ± 0.01 | 0.02 ± 0.00 | 4.10 ± 0.86 | 0.00 ± 0.00 |
| *T. suecica*  +  *Euplotes* sp. | 5 |  | n.d. | 521 ± 86.4 | 87.7 ± 5.28 | 2.48 ± 1.44 | 9.94 ± 2.93 | 1.33 ± 1.25 | 9.91 ± 6.35 | 0.00 ± 0.00 | 0.05 ± 0.06 | 0.85 ± 0.65 | 0.00 ± 0.00 | 0.01 ± 0.00 | 0.09 ± 0.04 | 0.02 ± 0.01 | 11.0 ± 14.6 | 0.00 ± 0.00 |
|  | 30 |  | n.d. | 435 ± 30.1 | 63.9 ± 12.3 | 4.94 ± 2.73 | 9.64 ± 4.74 | 1.23 ± 1.00 | 13.6 ± 8.04 | 0.00 ± 0.00 | 0.06 ± 0.03 | 0.74 ± 0.33 | 0.00 ± 0.00 | 0.01 ± 0.00 | 0.12 ± 0.04 | 0.01 ± 0.00 | 14.8 ± 9.20 | 0.00 ± 0.00 |
| *T. suecica*  *+*  *A. tonsa* | 5 |  | n.d. | 434 ± 29.2 | 80.7 ± 6.11 | 3.92 ± 0.48 | 5.44 ± 0.85 | 0.13 ± 0.09 | 1.90 ± 0.25 | 0.00 ± 0.00 | 0.02 ± 0.01 | 1.99 ± 0.58 | 0.00 ± 0.00 | 0.01 ± 0.00 | 0.07 ± 0.01 | 0.01 ± 0.00 | 0.53 ± 0.26 | 0.01 ± 0.00 |
|  | 30 |  | n.d. | 443 ± 19.1 | 83.3 ± 5.51 | 4.00 ± 0.55 | 5.39 ± 1.64 | 0.04 ± 0.02 | 1.91 ± 0.53 | 0.02 ± 0.01 | 0.03 ± 0.02 | 3.21 ± 0.32 | 0.00 ± 0.00 | 0.01 ± 0.00 | 0.08 ± 0.03 | 0.01 ± 0.01 | 0.91 ± 1.02 | 0.01 ± 0.00 |
|  |  |  |  |  |  |  |  |  |  |  |  |  |  |  |  |  |  |  |
| *T. weissflogii* | 5 |  | 69.6 ± 29.2 | 390 ± 29.3 | 55.1 ± 5.71 | 4.78 ± 2.00 | 6.12 ± 2.56 | 1.73 ± 0.72 | 0.99 ± 0.43 | 0.00 ± 0.00 | 0.08 ± 0.03 | 2.03 ± 0.85 | 0.00 ± 0.00 | 0.01 ± 0.00 | 0.06 ± 0.02 | 0.10 ± 0.04 | 0.04 ± 0.02 | 0.01 ± 0.00 |
|  | 30 |  | 59.4 ± 16.02 | 1194 ± 14.05 | 53.6 ± 4.69 | 2.60 ± 0.71 | 6.84 ± 1.84 | 1.47 ± 0.40 | 0.88 ± 0.24 | 0.01 ± 0.00 | 0.09 ± 0.02 | 3.06 ± 0.83 | 0.01 ± 0.00 | 0.01 ± 0.00 | 0.06 ± 0.02 | 0.09 ± 0.03 | 0.04 ± 0.01 | 0.01 ± 0.00 |
| *T. weissflogii*  +  *Euplotes* sp. | 5 |  | 57.7 ± 0.00 | 365 ± 48.7 | 59.8 ± 3.15 | 3.74 ± 1.42 | 7.22 ± 0.00 | 1.09 ± 0.00 | 2.28 ± 0.00 | 0.00 ± 0.00 | 0.08 ± 0.00 | 2.08 ± 0.00 | 0.00 ± 0.00 | 0.01 ± 0.00 | 0.10 ± 0.00 | 0.07 ± 0.00 | 1.17 ± 0.00 | 0.01 ± 0.00 |
|  | 30 |  | 64.2 ± 0.00 | 376 ± 20.1 | 57.5 ± 2.37 | 3.65 ± 0.00 | 6.80 ± 0.00 | 1.42 ± 0.00 | 1.75 ± 0.00 | 0.00 ± 0.00 | 0.01 ± 0.00 | 2.19 ± 0.00 | 0.00 ± 0.00 | 0.01 ± 0.00 | 0.11 ± 0.00 | 0.05 ± 0.00 | 1.28 ± 0.00 | 0.01 ± 0.00 |
| *T. weissflogii*  +  *A. tonsa* | 5 |  | 80.5 ± 11.4 | 447 ± 33.0 | 65.0 ± 7.21 | 9.31 ± 3.35 | 11.3 ± 2.88 | 0.87 ± 0.63 | 1.07 ± 0.21 | 0.00 ± 0.00 | 0.07 ± 0.01 | 3.26 ± 0.37 | 0.00 ± 0.00 | 0.01 ± 0.00 | 0.15 ± 0.02 | 0.65 ± 1.25 | 0.07 ± 0.12 | 0.01 ± 0.00 |
|  | 30 |  | 25.5 ± 3.04 | 94.6 ± 9.53 | 15.2 ± 0.70 | 3.41 ± 1.52 | 2.83 ± 0.25 | 0.29 ± 0.16 | 0.59 ± 0.34 | 0.00 ± 0.00 | 0.03 ± 0.00 | 1.23 ± 0.29 | 0.00 ± 0.00 | 0.00 ± 0.00 | 0.04 ± 0.00 | 0.05 ± 0.06 | 0.01 ± 0.00 | 0.00 ± 0.00 |
|  |  |  |  |  |  |  |  |  |  |  |  |  |  |  |  |  |  |  |
| *Synechococcus* sp. | 5 |  | n.d. | 395 ± 18.3 | 74.0 ± 8.89 | 19.2 ± 3.30 | 4.34 ± 0.82 | 1.26 ± 0.22 | 1.92 ± 0.33 | 0.01 ± 0.00 | 0.04 ± 0.01 | 4.55 ± 0.79 | 0.00 ± 0.00 | 0.01 ± 0.00 | 0.05 ± 0.01 | 0.01 ± 0.00 | 0.12 ± 0.02 | 0.03 ± 0.01 |
|  | 30 |  | n.d. | 399 ± 14.9 | 76.9 ± 6.43 | 19.9 ± 5.68 | 5.44 ± 1.55 | 1.15 ± 0.33 | 1.31 ± 0.37 | 0.05 ± 0.01 | 0.05 ± 0.01 | 5.67 ± 1.61 | 0.01 ± 0.00 | 0.01 ± 0.00 | 0.08 ± 0.02 | 0.01 ± 0.00 | 0.10 ± 0.03 | 0.04 ± 0.01 |
| *Synechococcus* sp.  +  *Euplotes* sp. | 5 |  | n.d. | 414 ± 16.6 | 91.5 ± 4.34 | 4.97 ± 5.37 | 1.36 ± 1.47 | 1.37 ± 1.52 | 8.02 ± 10.8 | 0.00 ± 0.00 | 0.01 ± 0.00 | 1.58 ± 0.62 | 0.00 ± 0.00 | 0.03 ± 0.03 | 0.08 ± 0.07 | 0.05 ± 0.02 | 0.19 ± 0.13 | 0.01 ± 0.00 |
|  | 30 |  | n.d. | 411 ± 37.4 | 83.2 ± 6.02 | 7.72 ± 5.15 | 4.74 ± 0.57 | 0.87 ± 0.39 | 2.36 ± 1.33 | 0.00 ± 0.00 | 0.00 ± 0.00 | 0.35 ± 0.30 | 0.00 ± 0.00 | 0.01 ± 0.00 | 0.03 ± 0.01 | 0.08 ± 0.07 | 0.08 ± 0.05 | 0.01 ± 0.01 |
| *Synechococcus* sp.  +  *A. tonsa* | 5 |  | n.d. | 405 ± 35.3 | 60.0 ± 5.66 | 5.94 ± 2.04 | 2.80 ± 0.80 | 0.09 ± 0.00 | 0.62 ± 0.36 | 0.00 ± 0.00 | 0.04 ± 0.01 | 2.67 ± 0.21 | 0.00 ± 0.00 | 0.01 ± 0.00 | 0.05 ± 0.00 | 0.01 ± 0.00 | 0.01 ± 0.00 | 0.00 ± 0.00 |
|  | 30 |  | n.d. | 97.3 ± 9.17 | 72.0 ± 18.5 | 28.5 ± 17.1 | 8.67 ± 2.45 | 0.56 ± 0.36 | 5.96 ± 3.43 | 0.09 ± 0.06 | 0.15 ± 0.08 | 40.7 ± 22.3 | 0.01 ± 0.01 | 0.04 ± 0.04 | 0.31 ± 0.24 | 0.03 ± 0.02 | 0.09 ± 0.03 | 0.02 ± 0.01 |
